# Recent advances in the pharmacology of voltage-gated ion channels

**DOI:** 10.1016/j.pharmr.2025.100090

**Published:** 2025-09-12

**Authors:** Diego Lopez-Mateos, Brandon John Harris, Adriana Hernández-González, Vladimir Yarov-Yarovoy, Heike Wulff

**Affiliations:** 1 Department of Physiology and Membrane Biology, School of Medicine, University of California Davis, Davis, California; 2 Department of Anesthesiology and Pain Medicine, School of Medicine, University of California Davis, Davis, California; 3 Department of Pharmacology, School of Medicine, University of California Davis, Davis, California

## Abstract

Voltage-gated ion channels (VGICs) are critical regulators of membrane potential, cellular excitability, and calcium signaling in both excitable and nonexcitable tissues and constitute important drug targets for neurological, cardiovascular, and immunological diseases. This review describes recent progress in the pharmacology of voltage-gated Na^+^, voltage-gated Ca^2+^, and voltage-gated K^+^ channels, highlighting clinical-stage compounds, emerging therapeutic modalities, and new strategies in VGIC drug discovery, emphasizing the increasingly central role of protein structures and artificial intelligence. Several compounds targeting VGICs have progressed to clinical trials for epilepsy, atrial fibrillation, psoriasis, and difficult-to-treat disorders, such as chronic pain, schizophrenia, major depression, and amyotrophic lateral sclerosis. The therapeutic landscape for VGIC-related disorders is expanding beyond traditional small molecules and antisense oligonucleotides and gene therapies targeting VGICs at the mRNA or gene level are currently in both early and late clinical trial stages for Dravet syndrome and developmental epileptic encephalopathy. The progression of such varied modalities suggests that the extensive efforts dedicated to elucidating VGIC biophysics and structure, coupled with rigorous target validation, are beginning to translate into therapeutic advancements. Furthermore, we discuss emerging discovery strategies, including the growing impact of VGIC structures, computational structural modeling, virtual screening of focused and ultralarge libraries, and artificial intelligence–driven redesign and de novo design of biologics. Although these approaches are poised to substantially accelerate the early stages of ion channel drug discovery, the clinical stages will continue to require careful selection of indications and thoughtful clinical trial design to fully realize the long-held potential of VGICs as drug targets.

**Significance Statement:**

Drug development for voltage-gated ion channels is widely considered to be challenging. This article reviews recent advances in the pharmacology of voltage-gated Na^+^, voltage-gated Ca^2+^, and voltage-gated K^+^ channels by examining compounds currently in clinical trials, including emerging new therapeutic approaches such as antisense oligonucleotides and gene therapy. We then discuss noteworthy recent developments, including the increasing availability and impact of ion channel structures, structural modeling, virtual screening, and artificial intelligence-assisted protein design, which are likely to accelerate the early stages of ion channel drug discovery. Success of the later stages will continue to rely on rigorous target validation, and proper choices of clinical candidates and clinical trial design.

## Graphical abstract

**Figure:**
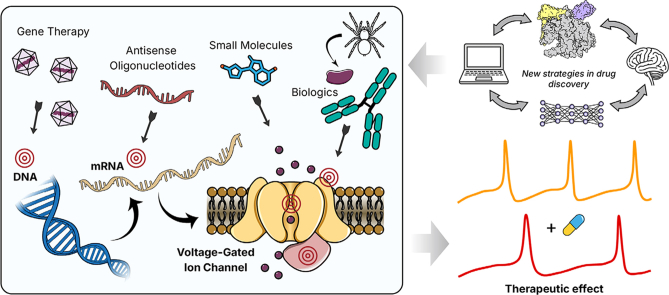

## I Introduction

**Fig. 1:**
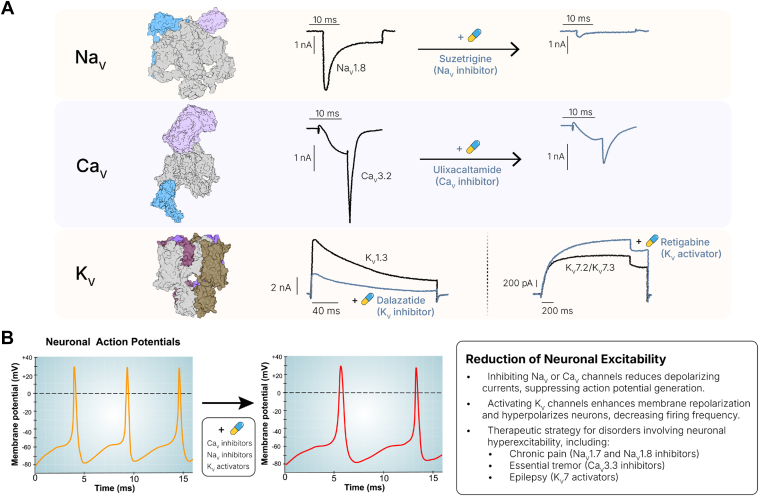
Pharmacological modulation of VGICs. (A) Representative current traces for each VGIC type illustrating the effects of drugs discussed in the main text. Traces were adapted from published studies: (1) Na_V_1.8 + Suzetrigine^2^; (2) Ca_V_3.2 + Ulixacaltamide^3^; (3) K_V_1.3 + Dalazatide^4^; (4) K_V_7.2/7.3 + Retigabine.^5^ (B) VGIC-targeting drugs can reduce neuronal hyperexcitability by inhibiting depolarizing Na_V_ and Ca_V_ currents or by activating repolarizing K_V_ currents. These mechanisms suppress or dampen action potential firing and are the basis of therapeutic strategies for disorders such as chronic pain, epilepsy, and ET. Examples of VGIC modulators relevant to this approach, in preclinical or clinical development, are discussed in the main text.

Voltage-gated ion channels (VGICs) conducting sodium, calcium, and potassium ions play fundamental roles in physiology and constitute important targets for analgesic, antiseizure, antiarrhythmic, and antihypertensive drugs (Fig. 1A).^1^, ^2^, ^3^, ^4^, ^5^ VGICs therefore are some of the most extensively studied proteins, and for several of the more popular channels, there is exquisitely detailed biophysical and structural information describing gating and permeation and sites for pharmacological modulation and a vast body of knowledge about association with human diseases.^6^, ^7^, ^8^, ^9^ An illustration of the intense interest in VGICs is that voltage-gated Na^+^ (Na_V_), voltage-gated Ca^2+^ (Ca_V_) and voltage-gated K^+^ (K_V_) channels typically have separate platform and poster sessions dedicated to them at the annual Biophysical Society meetings. However, despite these long-ongoing, intense academic efforts and extensive programs in the pharmaceutical industry dedicated to Na_V_, Ca_V_, and K_V_ channel drug discovery, very few new drugs targeting VGICs have successfully translated into the clinic in the last 25 years. Progress has been hampered by the need for exquisite subtype selectivity across highly homologous isoforms, safety issues from off-target activity, and limited central nervous system (CNS) penetration.

Most of the clinically used drugs targeting VGICs, such as the antihypertensive dihydropyridines (DHPs) inhibiting Ca_V_ channels or the Na_V_ channel blocking local anesthetics (eg, lidocaine) and antiseizure drugs (eg, phenytoin and carbamazepine) were developed between 1950 and 1995, often long before their molecular targets were identified and cloned.^10^ For example, out of the highly promising K_V_ channel modulator programs we reviewed in 2009,^11^ only 1 compound, the K_V_7 channel activator retigabine made it to market in 2011 but was later withdrawn because of side effects. Since our “pre-COVID pandemic” analysis^10^ in 2019, the only new drug targeting a VGIC was the much-anticipated Na_V_1.8 blocker suzetrigine, which was approved by the US Food and Drug Administration (FDA) at the end of January 2025 as a first-in-class nonopioid analgesic, to treat moderate-to-severe acute pain in adults after receiving “Breakthrough Therapy,” “Fast Track,” and “Priority Review” designations for the review process.^12^

With hindsight, the ion channel drug discovery “renaissance” that had been predicted in 2010—following the improvements in cloning, molecular biology, cell culture techniques, and high-throughput assays, including automated electrophysiology—failed to materialize.^13^ Ion channels did not become the “next G protein-coupled receptor (GPCRs)” a decade ago^14^ but instead continued to be “difficult” drug targets. Indeed, a comprehensive analysis of molecular drug targets published in 2017 found that out of the drugs targeting human proteins that were approved between 2001 and 2015, the proportion of drugs targeting ion channels had steadily decreased, whereas the number of new drug approvals for GPCRs had remained steady, and the number of approvals for kinase inhibitors had dramatically increased.^15^ The reasons why ion channels, especially Na_V_, Ca_V_, and K_V_ channels are challenging to target are typically attributed to difficulties associated with achieving subtype selectivity among family members with a high degree of homology, the technical challenges, the expertise necessary to run ion channel assays, and the absence of high-resolution structures enabling structure-based drug design or virtual screening (VS).^10^ However, we would like to posit here that instead of blaming the targets as undruggable, the reasons for the difficulties in translation into the clinic might be varied and multiple and include the drug discovery, development, and approval process being more lengthy, more expensive, and more complicated than in the past when many of the 27 ion channel drugs listed on the World Health Organization List of Essential Medicines were discovered and developed. Given that many Na_V_, Ca_V,_ and K_V_ channels have broad tissue distributions and play immensely important but often also nuanced roles in physiology and pathophysiology, it could be that VGICs need a more extended “incubation time” and more advanced techniques and tools to be studied, as well as champions who are willing to devote time and resources to these fascinating proteins.

**Fig. 2:**
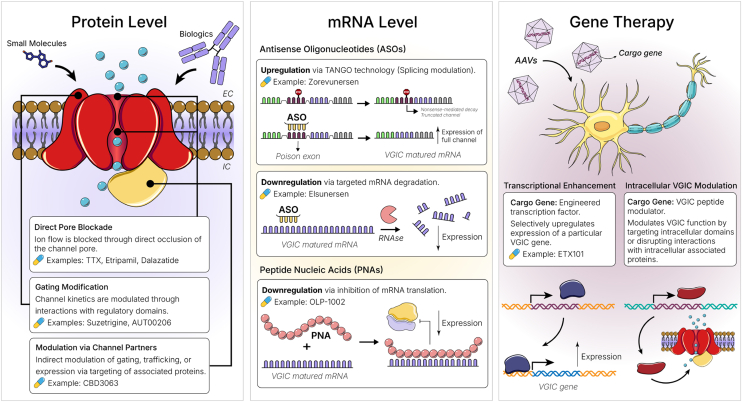
Therapeutic approaches for modulating VGICs. Current strategies under investigation target VGICs at 3 biological levels: protein level, mRNA level, and gene therapy. The examples shown here represent compounds in preclinical or clinical development, as discussed in the main text. AAV, adeno-associated virus; ASO, antisense olionucleotide; PNA, peptide nucleic acid; TANGO, targeted augmentation of nuclear gene output.

Although there certainly have been failures and twists and turns in VGICs modulator development, multiple small molecules inhibiting Na_V_1.1, Na_V_1.6, Na_V_1.8, and Ca_V_3.x channels or activating K_V_7.2/7.3 channels are currently in late-stage phase IIb or phase III clinical trials for pain, epilepsy, schizophrenia, and tremor. The therapeutic premise in all these cases is that Na_V_ or Ca_V_ channel inhibition suppresses action potentials, whereas K_V_ channel activation enhances membrane repolarization and hyperpolarizes neurons, leading to a reduction in neuronal excitability and firing frequency (Fig. 1B). Several other drug candidates including new exciting treatment modalities such as antisense oligonucleotides (ASOs) or gene therapies targeting VGICs not at the protein, but at the mRNA or gene level (Fig. 2), are both in early and late stages of clinical trials for various forms of epilepsy, including devastating childhood epilepsies such as Dravet syndrome and developmental epileptic encephalopathy (DEE), and difficult-to-treat disorders such as chronic pain, schizophrenia, major depression, atrial fibrillation, psoriasis, and amyotrophic lateral sclerosis (ALS). Table 1, Table 2, Table 3 show compounds targeting VGICs that are currently in clinical trials. Contemplating these compounds and their indications, it seems as if the many years of work devoted to understanding the biophysical properties and structure, including the beginning use of cryo-electron microscopy (cryo-EM) structures for drug design and thoughtful target validation using relevant animal models, human genetic data, and patient-derived tissues and cells, are starting to pay-off, and VGICs are beginning to live up to their therapeutic potential.

**Table 1:** **Figure:**
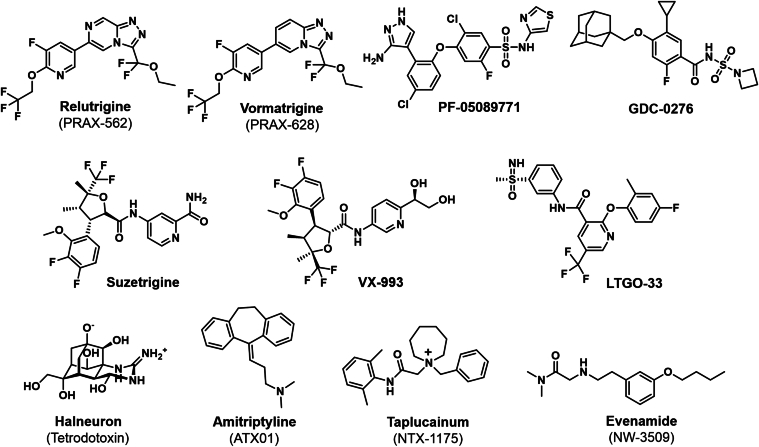

Structures of Na_V_ channel inhibitors and table listing compounds currently in clinical trials

Compound	Mechanism of Action	Indication	Company	Clinical Phase
ETX101	Na_V_1.1 gene therapy	Dravet syndrome	Encoded Therapeutics	Phase I/II
Relutrigine (PRAX-562)	Mixed Na_V_1.2/Na_V_1.6 blocker	SCN2A-DEE or SCN8A-DEE mutations	Praxis Precision Medicine	Phase II/III
Vormatrigine (PRAX-628)	Na_V_1.6 blocker	Focal onset seizures and generalized epilepsy	Praxis Precision Medicine	Phase II/III
Elsunersen (PRAX-222)	Na_V_1.2 ASO	SCN2A-DEE mutations	Praxis Precision Medicine	Phase II/III
Zorevunersen (STK-001)	Na_V_1.1 ASO	Dravet syndrome	Stoke Therapeutics	Phase III
OLP-1002	Na_V_1.7 peptide nucleic acid	Osteoarthritis pain	OliPass	Phase II
Suzetrigine (VX-548)	Na_V_1.8 blocker	Acute pain	Vertex Pharmaceuticals	FDA approval as JOURNAVX on January 30, 2025
		Chronic pain	Vertex Pharmaceuticals	Phase III
VX-993	Na_V_1.8 blocker	Acute pain	Vertex Pharmaceuticals	Phase II (completed June 26, 2025); failed
LTG-001	Na_V_1.8 blocker	Acute pain after abdominoplasty	Latigo Biotherapeutics	Phase II
LTG-001	Na_V_1.8 blocker	Acute pain after bunionectomy	Latigo Biotherapeutics	Phase II
LTG-305	Na_V_1.8 blocker	Chronic pain	Latigo Biotherapeutics	Phase I
HBW-004285	Na_V_1.8 blocker	Pain	Hyperway	Phase II
STC-004	Na_V_1.8 blocker	Pain	SiteOne	Phase I
Halneuron (TTX)	Na_V_1.x blocker	Chemotherapy-induced neuropathic pain	Dogwood Therapeutics	Phase IIb
ATX01	Na_V_1.7, Na_V_1.8 and Na_V_1.9 blocker	Topical for erythromelalgia	AlgTX	Phase II
		Topical for diabetic and Covid-induced neuropathy		Phase II
Taplucainium (NTX-1175)	Na_V_1.x blocker	Chronic cough	Nocion Therapeutics	Phase I
Evenamide (NW-3509)	Na_V_1.x blocker	Schizophrenia	Newron Pharmaceuticals	Phase III

ASO, antisense oligonucleotide; DEE, developmental epileptic encephalopathy.

**Table 2:** **Figure:**
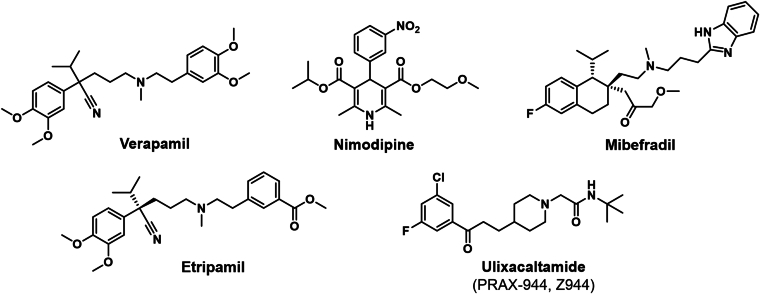

Structures of Ca_V_ channel inhibitors and table listing compounds currently in clinical trials

Compound	Mechanism of Action	Indication	Company	Clinical Phase
Etripamil	Ca_V_1.x blocker	PSVT	Milestone Pharma	New drug application submitted for CARDAMYST
		Atrial fibrillation		Phase III
Ulixacaltamide (PRAX-944)	Ca_V_3.x blocker	ET	Praxis Precision Medicine	Phase III

ET, essential tremor; PSVT, paroxysmal supraventricular tachycardia.

**Table 3:** **Figure:**
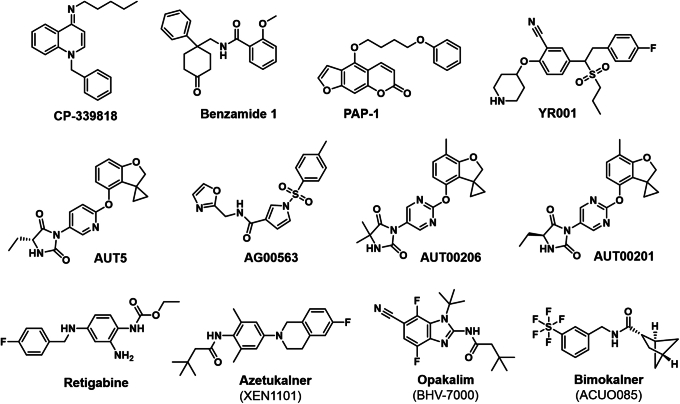

Structures of K_V_ channel modulators and table listing compounds currently in clinical trials

Compound	Mechanism of Action	Indication	Company	Clinical Phase
LY3972406 (DES-7114)	K_V_1.3 blocker	Plaque psoriasis	Eli Lilly	Phase II
si-544	K_V_1.3 blocker (peptide)	Psoriasis and psoriatic arthritis	selectION	Phase Ib
YR001	K_V_1.3 blocker	Topical for atopic dermatitis	Hangzhou Yirui Pharmaceutical Technology	Phase II
AUT00206	K_V_3.1/3.2 PAM	Fragile X syndrome	Autifony	Phase II
AUT00201	K_V_3.1/3.2 PAM	Progressive myoclonic epilepsy	Autifony	Phase I
Azetukalner (XEN1101)	K_V_7.2/7.3 PAM	Focal onset and tonic-clonic seizures MDD	Xenon Pharmaceuticals	Phase III
				Phase III
Opakalim (BHV-7000)	K_V_7.2/7.3 PAM	Focal and generalized epilepsy	Biohaven	Phase III
		Bipolar disorder		Phase II
		MDD		Phase II
QRL-101	K_V_7.2/7.3 PAM	ALS	QurAlis	Phase I
XEN1120	K_V_7 PAM	Analgesic	Xenon Pharmaceuticals	Phase I
Bimokalner (ACOU085)	K_V_7.4 PAM	Cisplatin induced hearing loss	Acousia Therapeutics	Phase IIa

ALS, amyotrophic lateral sclerosis; MDD, major depressive disorder; PAM, positive allosteric modulator.

In this article, we will review recent advances in the pharmacology of Na_V_, Ca_V,_ and K_V_ channels by examining compounds currently in clinical trials and then discuss novel developments, including the increasing impact of ion channel structures, structural modeling, VS, and artificial intelligence (AI)–assisted protein design, which are likely to accelerate the early stages of ion channel drug discovery. Readers interested in ion channel families and nomenclature are referred to the International Union of Basic and Clinical Pharmacology *Guide to Pharmacology*,^1^ which currently lists 145 human genes for voltage-gated–like ion channels,^16^ 82 human genes for ligand gated ion channels,^17^ and 52 human genes for aquaporins, connexins, and store-operated channels.^18^ Readers interested in the history of ion channel research and ion channel pharmacology are encouraged to read Bertil Hille’s iconic textbook^19^ and many excellent reviews,^7^^,^^20^^,^^21^ including a recently published book entitled “Ion Channels as Targets in Drug Discovery” edited by Gary Stephens and Edward Stevens.^22^

## II Recent advances in targeting voltage-gated Na^+^ channels

Na_V_ channels are crucial for action potential initiation and propagation in excitable cells. The 9 Na_V_ subtypes (Na_V_1.1–Na_V_1.9) in humans have distinct tissue expression patterns and physiological functions.^1^^,^^23^^,^^24^ Given their central role in excitability, Na_V_ channels are attractive targets for drug development, especially for conditions like pain, epilepsy, and cardiac arrhythmias. Classical small-molecule Na_V_ blockers, such as local anesthetics and antiepileptics, are currently used in the clinic but typically lack subtype selectivity, leading to off-target effects. Human genetic studies, such as *SCN9A* (Na_V_1.7) loss-of-function (LOF) mutations causing pain insensitivity and gain-of-function (GOF) mutations causing chronic pain, stimulated efforts to develop selective Na_V_ modulators.^24^, ^25^, ^26^, ^27^, ^28^, ^29^, ^30^ However, early translation of isoform-selective inhibitors proved challenging: many Na_V_1.7-selective drug candidates showed potent target engagement in vitro yet failed to produce analgesia in clinical trials.^31^ These insights have prompted new strategies in Na_V_ pharmacology to achieve effective and tissue-selective modulation, including targeting other Na_V_ channel subtypes, broad-spectrum Na_V_ channel blockers, ASO-based therapies, and gene-based therapies (Fig. 2 and Table 1). Since a decade ago, when small molecules dominated, we now have diverse modalities enabling tailored treatments for specific Na_V_ subtype dysfunctions. Notably, many of these modalities aim for disease modification, not just symptom management, offering the potential for long-term remission in chronic pain and epilepsy.

### A Precision medicine approaches to target voltage-gated Na^+^ channels in epilepsy and neurodevelopmental disorders

The pipeline of novel Na_V_ channel modulators (Table 1) consists mostly of therapies for specific channelopathies caused by either LOF or GOF mutations in Na_V_ channels such as Dravet syndrome (*SCN1A* [Na_V_1.1]), early-onset epilepsy (*SCN2A* [Na_V_1.2]), and *SCN8A* (Na_V_1.6)-related encephalopathy. The complexity of these disorders, with some patients requiring Na_V_ channel inhibition and others needing upregulation of Na_V_ channel expression, underscores the adaptability of these new therapeutic approaches. The hope is that these targeted therapies will not only reduce seizures but also positively impact associated symptoms like developmental delay and autistic features.

#### 1 Restoring Na_V_1.1 function in Dravet syndrome using antisense oligonucleotides and gene therapy

Dravet syndrome, which results from Na_V_1.1 LOF in inhibitory neurons, necessitates therapies that restore Na_V_1.1 function.^32^^,^^33^ STK-001 (trade name Zorevunersen) from Stoke Therapeutics is an ASO designed to selectively increase Na_V_1.1 protein levels using the targeted augmentation of nuclear gene output technology (Fig. 2), which prevents the inclusion of a specific “poison exon” in SCN1A pre-mRNA.^34^ Since poison exons introduce premature stop codons and trigger nonsense-mediated decay or result in truncated protein forms, STK-001 promotes the generation of full-length Na_V_1.1 from the healthy allele restoring functional protein levels.^35^ Preclinical studies in mice demonstrated the efficacy of STK-001, including seizure reduction and increased survival.^35^ Notably, STK-001 does not overexpress Na_V_1.1 above normal levels, minimizing potential toxicity.^35^ Phase II clinical trials have demonstrated significant and sustained seizure reduction and improvements in cognitive function in patients (NCT04442295 and NCT04740476). STK-001 received FDA Breakthrough designation and is currently undergoing phase III trials.

Encoded Therapeutics developed ETX101, a gene therapy approach, that has potential for a 1-time treatment for SCN1A+ Dravet syndrome.^33^^,^^36^ ETX101 utilizes an adeno-associated virus (AAV) serotype 9 vector to deliver an engineered transcription factor (Fig. 2) that selectively upregulates Na_V_1.1 expression in GABA-ergic interneurons, minimizing the risk of overexpression in other cell types. Preclinical studies demonstrated significant seizure reduction and improved survival in Dravet mice.^34^^,^^36^ Safety studies of ETX101 in primates demonstrated robust transgene expression throughout the brain, with no serious adverse effects. ETX101 received Investigational New Drug clearance in the United States and phase I/II clinical trials were initiated in infants and young children with Dravet syndrome (NCT06283212, NCT06112275, and NCT05419492).

#### 2 Targeting Na_V_1.2 and Na_V_1.6 in developmental epileptic encephalopathies

DEEs resulting from GOF mutations in *SCN2A* (Na_V_1.2) or *SCN8A* (Na_V_1.6) in excitatory neurons require treatments that reduce neuronal hyperexcitability. Relutrigine (PRAX-562), a compound developed by Praxis Precision Medicines, specifically targets the enhanced persistent sodium current that is observed in *SCN2A* (Na_V_1.2) or *SCN8A* (Na_V_1.6) GOF mutations.^37^ Relutrigine has potential to reduce neuronal hyperexcitability while minimizing the disruption of normal Na_V_1.2 or Na_V_1.6 channel function. Preclinical studies in DEE mouse models demonstrated robust seizure control. With a favorable safety profile established in phase I trials, relutrigine has advanced to phase II clinical trials in children with *SCN2A*-DEE and *SCN8A*-DEE (NCT05818553). The FDA has granted relutrigine Orphan and Rare Pediatric Disease designations. In parallel, Praxis Precision Medicines is developing the ASO elsunersen (PRAX-222) for early-onset *SCN2A*-DEE.^38^ Elsunersen lowers Na_V_1.2 protein expression by binding to *SCN2A* mRNA and triggering its degradation (Fig. 2). Preclinical studies of elsunersen revealed significant reductions in Na_V_1.2 expression and subsequent improvements in dose-dependent seizure reduction, improved behavior, and extended survival in *SCN2A* mutant mouse models. Elsunersen is administered intrathecally but requires periodic dosing to maintain therapeutic effects.^38^ A phase I/II trial is ongoing to assess safety and efficacy of elsunersen in children with *SCN2A*-DEE (NCT05737784). Since elsunersen affects both mutant and wild-type Na_V_1.2, careful dosing will be critical to avoid excessive Na_V_1.2 suppression below the level needed for healthy neuronal function.

In addition to the mixed Na_V_1.2/Na_V_1.6 blocker relutrigine (PRAX-562), Praxis Precision Medicine is also developing the Na_V_1.6-selective vormatrigine (PRAX-628) for adult focal and generalized epilepsy and for *SCN8A*-related pediatric epilepsies.^33^ GOF mutations in *SCN8A* can lead to severe early-onset epileptic encephalopathy (EE) and movement disorders. Preclinical studies demonstrated higher selectivity and potency of vormatrigine compared with standard anticonvulsants, with a wide therapeutic margin observed in phase I clinical trials. According to the company website, ongoing phase II/III trials are evaluating vormatrigine’s efficacy in refractory focal epilepsy (trial registration numbers are not available).

### B Lessons learned from targeting Na_V_1.7 for pain

As mentioned above, Na_V_1.7 gained prominence as a key pain modulator based on human genetic syndromes, where LOF led to pain insensitivity and GOF caused chronic pain.^28^^,^^39^ These findings sparked intense efforts in industry and academia to develop Na_V_1.7-selective blockers.^40^ However, clinical trials of potent and selective small molecules targeting hNa_V_1.7 from Pfizer and Genentech failed because of insufficient target engagement, binding to serum albumin, and/or toxicity.^29^^,^^30^

#### 1 Small molecule Na_V_1.7 blockers

The aryl-sulfonamide PF-05089771 developed by Pfizer is the only Na_V_1.7 inhibitor to reach clinical trials, where it failed due to the lack of efficacy, presumably due to poor target engagement.^41^ Notably, aryl-sulfonamide compounds of this class are highly (>99.99%) and tightly bound to plasma proteins, which results in an extremely low fraction of free drug available for target engagement with Na_V_1.7.^42^ New generation aryl-sulfonamide Na_V_1.7 inhibitors with zwitterionic groups, such as GDC-0276 developed by Genentech, achieved a higher level of target engagement and efficacy in pain models.^43^ However, a phase I trial of GDC-0276 revealed liver toxicity and a decrease in blood pressure.^44^ Blood pressure decreases were also observed in safety studies in nonhuman primates with ST-2560 developed by SiteOne Therapeutics, another highly selective Na_V_1.7 blocker, suggesting the involvement of Na_V_1.7 channels in autonomic ganglia in regulating heart rate and blood pressure.^45^ Interestingly, autonomic function, including blood pressure, is normal in patients with LOF Na_V_1.7 mutations. Furthermore, a recent Genentech study demonstrated that Na_V_1.7 function is required for the initiation of C-fiber action potentials, which explains the observed insensitivity to pain following genetic removal or inhibition of Na_V_1.7.^46^ We believe that it might be possible to avoid “on target” blood pressure lowering side effects of Na_V_1.7 blockers with repeated lower doses or slow-release formulations of Na_V_1.7 inhibitors^42^ or by using gene-based interventions to replicate the LOF phenotype observed in humans.^47^ For patients with severe pain conditions, intrathecal administration could be another way to avoid blood pressure lowering effects.

#### 2 Knockdown approaches targeting Na_V_1.7

OLP-1002, a peptide nucleic acid, developed by the South Korean drug company OliPass, inhibits *SCN9A* mRNA translation (Fig. 2) to replicate the pain-free phenotype observed in humans with *SCN9A* (Na_V_1.7) LOF mutations.^48^ Localized delivery of OLP-1002 resulted in significant and sustained pain relief in a phase IIa clinical trial (NCT05216341). Another innovative strategy involves AAV-mediated gene therapy, delivering a peptide that disrupts Na_V_1.7-cytosolic collapsin response mediator protein 2 (CRMP2) interactions (AAV-Na_V_1.7-CRS), effectively “dialing down” Na_V_1.7 function.^49^ Preclinical studies of AAV-Na_V_1.7-CRS showed rapid and sustained pain relief in mouse models of chronic pain.^49^

### C A breakthrough in nonopioid analgesia with Na_V_1.8 blockers

Vertex Pharmaceuticals developed suzetrigine (VX-548), a highly potent and selective small molecule that binds to the Na_V_1.8 voltage-sensing domain II (VSD-II) in a deactivated state to stabilize a closed state of the channel.^2^^,^^50^ Suzetrigine achieved substantial pain reduction compared with placebo, with efficacy comparable with hydrocodone and minimal adverse events in phase II clinical trials for acute postoperative pain following bunionectomy and abdominoplasty surgery.^50^ These results demonstrate the first clinical validation of a subtype-selective Na_V_ inhibitor for analgesia. Recently, suzetrigine (JOURNAVX) has been FDA-approved as a first-in-class nonopioid analgesic, offering an effective and safe alternative to opioids.^51^ Vertex Pharmaceuticals is advancing follow-on small molecules, such as VX-993, to further enhance efficacy and safety. However, VX-993 recently failed to differentiate itself from placebo after bunion removal surgery in a phase II trial and therefore will not be advanced further as a monotherapy for acute pain.

Latigo Biotherapeutics developed 2 other promising small molecules, LTG-001 and LTG-305, targeting Na_V_1.8 for chronic pain. These compounds, derived from a rational design approach, target VSD-II to stabilize the closed state of Na_V_1.8 as described for the tool compound LTGO-33.^52^ LTG-001 was well tolerated with rapid absorption in phase I trial (NCT06049095) and received Fast Track Designation from the FDA in March 2025, advancing it to a phase II trial (NCT06774625). LTG-305 has recently started phase I (NCT06554574). Hyperway Pharma, a drug company in China, is developing HBW-004285, an oral Na_V_1.8 inhibitor in phase II clinical trials, according to the company website, highlighting the global focus on this target. Another Na_V_1.8 inhibitor that recently completed phase I studies with promising results, according to the company’s website, is STC-004 from SiteOne Therapeutics (recently acquired by Eli Lilly), which has been developed with funding from the National Institutes of Health Helping to End Addiction Long-term Initiative program.

### D Broad-spectrum voltage-gated Na^+^ blockers and toxins for pain

Broad-spectrum Na_V_ channel blockers, such as tetrodotoxin (TTX), represent an alternative approach to isoform-selective Na_V_ inhibition. This potent toxin, which blocks most Na_V_ subtypes except Na_V_1.5, Na_V_1.8, and Na_V_1.9, effectively reduces hyperexcitability of peripheral neurons.^53^^,^^54^ Administered subcutaneously or intramuscularly due to its poor oral bioavailability, TTX can exert a peripherally restricted analgesia, minimizing CNS side effects.^55^ An underpowered phase III clinical trial conducted by WEX Pharmaceuticals in 2017 demonstrated clinically meaningful analgesia for twice daily subcutaneous TTX in moderate-to-severe cancer-related pain, although side effects like perioral numbness were observed.^56^ Dogwood Therapeutics, a new company resulting from the merger of WEX with 2 other biotech companies, recently received FDA Fast Track designation for its TTX formulation named Halneuron and initiated a new phase IIb clinical trial for chemotherapy-induced neuropathy (NCT06848348).

Another interesting approach is localized peripheral nerve blockade with ATX01, a topical amitriptyline formulation developed by AlgoTherapeutix, which broadly inhibits Na_V_1.7, Na_V_1.8, and Na_V_1.9 channels.^57^ This high-strength dermal gel aims to deliver therapeutic concentrations of amitriptyline directly to cutaneous nociceptors, minimizing systemic exposure and associated CNS side effects. Although a phase II trial for chemotherapy-induced peripheral neuropathy showed promising pain reduction with ATX01 (NCT05593614), high placebo responses at certain sites obscured overall statistical significance. However, post-hoc analysis of a subset of sites revealed significant pain relief.^57^ Importantly, ATX01 was well tolerated, supporting the concept of a peripherally restricted Na_V_ blocker for localized analgesia. ATX01 received Orphan Drug designation for erythromelalgia and is also being tested in phase II trials for diabetic and post-COVID neuropathic pain.

### E Novel uses of voltage-gated Na^+^ channel blockers in psychiatry

Beyond traditional applications in pain and epilepsy, Na_V_ channel modulation is also being explored in psychiatry, with evenamide (NW-3509), being developed by Newron Pharmaceuticals (Table 1). Evenamide inhibits several Na_V_ channels, including Na_V_1.3, Na_V_1.7, and Na_V_1.8, and modulates glutamate release,^58^ offering a novel approach to treating treatment-resistant schizophrenia. By dampening Na_V_ channel-dependent firing without affecting baseline neurotransmission,^58^ evenamide is assumed to normalize hyperexcitable glutamatergic neurons, hypothesized to contribute to persistent positive symptoms in treatment-resistant schizophrenia. Preclinical and early-phase clinical trials have shown promising results, including reduced aberrant glutamate spikes, antipsychotic-like effects, and significant symptom improvement in patients with schizophrenia. Following completion of phase II trials (NCT04461119), evenamide is currently in phase III trials and could become the first Na_V_ channel blocker approved for a psychiatric indication.

### F Emerging therapeutic strategies for voltage-gated Na^+^ channels

There is an increasing interest in targeting Na_V_ channels with biologics such as antibodies and venom peptides,^10^ although they have not yet progressed to clinical trials. Monoclonal antibodies (mAbs) have the potential for high potency and selectivity by binding to unique extracellular epitopes on Na_V_ channels. For example, mAbs could function as allosteric inhibitors, stabilizing a voltage-sensor domain in a deactivated state to prevent channel opening. Recently, bispecific antibodies and ligand-antibody conjugates engineered using a biepitopic crosslinking strategy have demonstrated potency and selectivity for Na_V_1.7.^59^ Subcutaneous delivery of anti-Na_V_1.7 mAbs could provide localized pain relief. mAbs targeting Na_V_ channels hold promise for long-acting, monthly dosing pain relief, similar to those targeting CGRP in migraine prevention.^60^

Venom peptides could serve as another structural template for the development of novel pain therapeutics targeting Na_V_ channels. Protoxin-II and Huwentoxin-IV are peptide toxins that potently and moderately selectively bind to the Na_V_1.7 VSD-II in a deactivated state, inspiring the development of more potent and selective peptide-based modulators.^61^^,^^62^ Additionally, mRNA therapeutics encoding therapeutic peptides offer a promising approach for transient expression and reduced dosing frequency.^63^^,^^64^

## III Recent advances in voltage-gated Ca^2+^ channel pharmacology and emerging therapeutics

Ca_V_ channels mediate Ca^2+^ influx in response to membrane depolarization and play essential roles in a wide range of physiological processes, including action potential firing, neurotransmitter release, muscle contraction, and gene expression.^7^ Ca_V_ channels are broadly classified into 3 families based on electrophysiological and molecular properties: Ca_V_1 (L-type) channels, which trigger excitation-contraction coupling in cardiac, skeletal, and smooth muscle and hormone secretion in endocrine cells; Ca_V_2 (P/Q-, N-, and R-type) channels, predominantly found in neurons mediating fast synaptic transmission, and Ca_V_3 (T-type) channels, which support pacemaker activity of the sinoatrial node and repetitive firing of action potentials in cardiac muscle and neurons.^1^^,^^7^ Ca_V_ channels have long been attractive targets in pharmacology, with classic L-type channel blockers used for treatment of hypertension and arrhythmias, N-type blockers for pain, and T-type blockers for epilepsy.^23^ Lead candidates, such as etripamil and ulixacaltamide (Table 2), show that it is possible to overcome historical challenges with innovative design and trial methodologies. As these compounds progress toward approval, they could pave the way for a new generation of Ca_V_ channel-targeted therapies with applications in cardiology and neurology.

### A Efforts focused on voltage-gated Ca_V_1 channels as targets for cardiac arrhythmia

One of the most recent advances in Ca_V_ channel pharmacology is etripamil (CARDAMYST), developed by Milestone Pharma, a novel L-type calcium channel blocker formulated for intranasal delivery.^65^ Etripamil is a non-DHP Ca_V_1 channel blocker that is chemically similar to verapamil and designed to be ultra-short-acting and suitable for at-home self-administration as a nasal spray. Etripamil’s lead indication is emergency care for paroxysmal supraventricular tachycardia (PSVT), an episodic arrhythmia caused by reentrant circuits in the heart’s atrioventricular node.^65^^,^^66^ The innovative intranasal route enables etripamil to rapidly enter the systemic circulation and reach the heart within 7 minutes,^66^ producing a transient atrioventricular nodal blockade that interrupts the tachycardia allowing patients to self-manage PSVT episodes. In a phase III clinical trial (NCT03464019), etripamil converted a substantial fraction of PSVT episodes to normal sinus rhythm.^67^^,^^68^ In an open-label phase III clinical trial (NCT03635996) following a randomized trial, about 60% of patient-reported PSVT episodes were successfully terminated after etripamil administration.^69^ There were minimal side effects and no cases of profound hypotension or bradycardia requiring intervention. Milestone Pharma submitted a new drug application for CARDAMYST (etripamil) to the FDA in May 2024 and received a communication in March 2025 that did not raise any concerns regarding clinical safety or efficacy but requested that chemistry, manufacturing, and controls issues be addressed. The same concern about a nitrosamine impurity led to a more recent rejection of the revised new drug application, which will likely require an inspection of the synthesis facilities. The development of etripamil, which is also currently being evaluated in phase III for atrial fibrillation (NCT06716021), highlights how re-engineering known drugs (verapamil, in this case) can yield new clinical applications.

### B Efforts focused on voltage-gated Ca_V_3 channels for the treatment of essential tremor

The traditional T-type (Ca_V_3) channel blocker mibefradil (POSICOR) was unselective and potently blocked cytochrome P450 3A4, leading to its withdrawal from the market^70^ after reports of serious drug-drug interactions in 1998. Newer compounds like Z944,^3^^,^^71^ now called ulixacaltamide (Z944, PRAX-944) have been optimized for selectivity and tolerability. Although initially studied in rodent models of pain^72^^,^^73^ and absence seizures,^3^ ulixacaltamide is now being pursued as a therapy for essential tremor (ET) by Praxis Precision Medicine.^74^ ET is the most common movement disorder, characterized by involuntary shaking of the hands, head, or voice. The pathophysiology of ET is thought to involve oscillatory thalamocortical networks, in which Ca_V_3 channels play a role by generating low-threshold Ca^2+^ spikes. Ulixacaltamide is a potent and highly selective inhibitor of Ca_V_3 channels with a preference for Ca_V_3.3 and Ca_V_3.2 subtypes.^3^ In preclinical models, ulixacaltamide dramatically reduced tremor amplitude without causing sedation or ataxia. In a rat harmaline tremor model, the drug dose-dependently suppressed tremor at well tolerated doses, with no locomotion impairment.^74^ A key aspect of its development has been the use of electroencephalogram sigma power as a translational biomarker for Ca_V_3 channel engagement. Ca_V_3 channels in thalamic neurons contribute to sleep spindle (sigma) rhythms. Ulixacaltamide reduced sigma power in animals, and encouragingly, similar reductions in sigma power were observed in a phase I trial in healthy volunteers at doses that were well tolerated.^74^ Ulixacaltamide is currently in phase III trials (NCT06087276) for ET, making it one of the most advanced Ca_V_ channel therapeutics in development.

### C Emerging therapeutic strategies for voltage-gated Ca^2+^ channels

With high-resolution Ca_V_ channel structures now available, it has become possible to apply VS and rational design to identify compounds with high potency and selectivity for a specific Ca_V_ subtype. For example, CBD3063, a peptidomimetic that inhibits an intracellular regulatory interaction of the N-Type Ca_V_2.2 channel, was discovered through molecular modeling and VS.^75^^,^^76^ Ca_V_2.2 activity is regulated by a cytosolic protein (Fig. 2), CRMP2, which enhances channel expression and function. CBD3063 effectively suppressed channel activity by disrupting the Ca_V_2.2–CRMP2 coupling and demonstrated analgesic effects in preclinical neuropathic and inflammatory pain models.^75^^,^^76^ Notably, CBD3063 preserved normal pain sensitivity and did not produce sedative or depressive side effects in preclinical tests.^75^^,^^76^

Another creative way of targeting the T-type channel Ca_V_3.2 for pain relief is to reduce association with the deubiquitinase USP5, which stabilizes the channel and increases its surface expression, with cell-permeant peptides or small molecules that disrupt the enzyme/channel interface at the domain III–IV linker of Ca_V_3.2.^77^, ^78^, ^79^ Both approaches reduce pain in rodent models of inflammatory and neuropathic pain. One of the small molecule “disruptors” seems to have advanced to the stage of development candidate at the Canadian company Zymedyne Therapeutics and is projected to start phase I clinical trials in 2026 according to the company website.

## IV Recent progress in targeting voltage-gated K^+^ channels

By allowing K^+^ ions to flow out of cells, K_V_ channels play a key role in regulating membrane potential. The 40 K_V_ channels present in the human genome are arranged into 12 subfamilies^16^ and have been physiologically subdivided into A-type channels showing fast inactivation and delayed rectifier potassium channels which inactivate much more slowly.^80^ Based on their diverse tissue distribution and their often highly specialized roles, the various K_V_ channels have tremendous potential as drug targets for neurological and cardiovascular disorders as well as for cancer, autoimmune diseases, and metabolic disorders.^11^^,^^22^ In excitable cells such as neurons or cardiomyocytes, K_V_ channels are responsible for the repolarization after action potential firing. Inhibition of K_V_ channels delays repolarization and typically increases excitability of neurons and muscle, whereas activation of K_V_ channels has the opposite effect and decreases excitability.^11^ In both excitable and nonexcitable cells K_V_ channels further play an important role in Ca^2+^ signaling, volume regulation, secretion, proliferation and migration, and K_V_ channel inhibitors typically reduce cellular activation and associated processes. There are many reviews and databases on K_V_ channel pharmacology,^11^^,^^16^^,^^22^ and the liability associated with the unintended targeting of K_V_11.1 (hERG) due to drug-induced arrhythmias that interested readers can refer to.^81^^,^^82^ In the following paragraphs, we will focus on K_V_1.3 blockers and positive allosteric modulators (PAMs) for K_V_3 and K_V_7 channels, which are currently in clinical trials for autoimmune diseases, epilepsy, depression, and several other neurological disorders (Table 3).

### A K_V_1.3 as a target for immunomodulation

K_V_ channels with the biophysical properties of K_V_1.3 were first discovered in human T cells 40 years ago^83^^,^^84^ and proposed as potential targets for immunosuppression because of their role in regulating membrane potential and calcium signaling during T-cell activation.^85^^,^^86^ Because K_V_1.3 inhibitors can reduce calcium influx into T cells, they were initially viewed as a potentially less nephrotoxic replacement of the calcineurin inhibitor cyclosporine. Both Merck and Pfizer initiated small molecule K_V_1.3 programs in the mid-1990s, which resulted in the identification of several pharmacophores exemplified by Pfizer’s CP-339818 and UK-78282,^87^ and Merck’s triterpene correolide^88^ and benzamide series.^89^ However, for many reasons including off-target toxicity and lack of a sufficiently strong immunosuppressive effects of these early compounds, interest in K_V_1.3 inhibitors waned, but revived again around 2006 following reports that K_V_1.3 inhibitors preferentially affect the function of effector memory T cells associated with human autoimmune diseases and can treat rodent models of multiple sclerosis, autoimmune diabetes, and rheumatoid arthritis.^90^, ^91^, ^92^ Based on these findings, it was recognized that K_V_1.3 inhibitors are mild immunosuppressants, and therefore, they rather constitute immunomodulators for the treatment of T-cell mediated autoimmune diseases instead of general immunosuppressants for the prevention of transplant rejection.^93^ Several academic groups and biopharmaceutical companies subsequently developed and advanced K_V_1.3 inhibitors.

#### 1 Targeting K_V_1.3 with venom-derived peptides

Following extensive structure-activity studies to improve selectivity over the neuronal K_V_1.1 channel,^94^^,^^95^ George Chandy’s group progressed ShK-186 (dalazatide), a derivative of the sea anemone peptide ShK through Investigational New Drug–enabling toxicity studies,^95^^,^^96^ together with the now defunct company Kineta, and demonstrated efficacy in a small phase Ib study in patients with plaque psoriasis.^97^ At the higher dose of 60 *μ*g, ShK-186 reduced psoriasis score in 9 out of 10 patients and reduced plasma levels of multiple inflammation markers. The most common adverse events were mild paresthesias of the hands and feet, which could have been compound related.^97^ A planned phase II trial in the orphan disease inclusion body myositis^98^ never started enrolling because of a lack of funding. Other groups worked on improving the pharmacokinetic properties of K_V_1.3 blocking peptides through half-life prolonging modifications such as PEGylation,^99^ fusion to Fc-fragments,^100^ or albumin.^101^ Groups at Amgen and Janssen tested several of these modified ShK or OSK1 derivatives in nonhuman primates^102^ or minipigs.^101^ However, both companies subsequently dropped K_V_1.3 as a target after reporting that K_V_1.3 inhibition, while undoubtedly immunomodulatory, was not sufficiently immunosuppressive to warrant development and could be overcome by strong T-cell stimulation.^103^^,^^104^ Undeterred by these reports, the German company selectION Therapeutics advance si-544, a K_V_1.3 selective peptide identified with a phage display platform for modified venom peptides, finished a phase I safety study (NCT05383378) in 2023, and just completed a phase Ib study in 45 adults with psoriasis or psoriatic arthritis (NCT06191042), which has not yet posted results. The presence of K_V_1.3 on autoreactive effector memory T cells and its easy accessibility to biologics also motivated efforts to target K_V_1.3 with conventional mAbs,^105^ nanobodies,^106^ and so-called KnotBodies, an approach where potent K_V_1.3 blocking venom peptides like ShK or the scorpion toxin peptide Vm24 are engineered into the complementarity-determining regions loops of antibodies.^107^^,^^108^ However, so far, none of these K_V_1.3 targeting biologics^10^ seem to have advanced toward the clinic.

#### 2 Targeting K_V_1.3 with small molecules

On the small molecule side, academic groups at the University of California, Davis and University of Melbourne developed 2 natural product-derived compounds, the psoralen PAP-1 and a series of khellinones,^109^ which were recently reviewed in depths together with other small molecule K_V_1.3 blockers including novel derivatives of the benzamides and mitochondrially targeted psoralen derivatives.^110^ Our own compound, PAP-1,^111^ was licensed by Circassia and tested in a phase I study in 2013 (NCT01743118) as a topical in comparison to placebo and to a marketed topical vitamin-D analog ointment in a 12-day long psoriasis plaque test, where ointment was applied to a 1 cm^2^ large area of skin. The study results were not encouraging presumably because the treatment period might have been too short, the area of skin covered by drug too small, or because PAP-1 did not penetrate sufficiently deep into the skin. The khellinone-type K_V_1.3 blockers were further optimized by the Australian Biotech company Bionomics, which entered into an agreement with Merck-Serono to develop this class of compounds for MS in 2008, but the agreement was ended in 2012 and the program abandoned. However, two structurally novel small molecule K_V_1.3 blockers advanced to phase II clinical trials. Eli Lilly tested the K_V_1.3 inhibitor LY3972406 as an oral for moderate to severe plaque psoriasis in a recently completed study that has not yet posted results (NCT06176768). This compound, which was previously called DES-7114, was licensed in May 2022 from DE Shaw Research, a company that uses their Anton supercomputers for drug discovery. Scientists at DE Shaw seem to have used molecular dynamics (MD) simulations^112^^,^^113^ to identify novel binding pockets in the K_V_1.3 protein that become accessible during conformational changes and developed a selective K_V_1.3 blocker compound by working with contract research organizations. Although the structure of DES-7114 has not been disclosed it has been published that the compound ameliorates inflammation in a humanized mouse model of ulcerative colitis.^114^ Another apparently K_V_1.3 targeting compound in a currently recruiting phase II clinical trial as an ointment for mild-to-moderate atopic dermatitis (NCT06309355) is YR001 from the Chinese company Hangzhou Yirui Pharma.

### B K_V_3 channel activation to reduce neuronal excitability in epilepsy, ataxia, and schizophrenia

K_V_3 family channels are encoded by the *KCNC1*-*KCNC4* genes and have been termed “enablers of rapid firing, neurotransmitter release, and neuronal endurance” because of their rapid activation and deactivation kinetics, allowing certain neurons to fire action potentials and to release neurotransmitters at rates of up to 1000 Hz.^115^ K_V_3 channels are expressed in fast-spiking GABAergic interneurons, cerebellar Purkinje cells, and auditory brain stem neurons.^115^ Mutations in K_V_3.1 have been reported to cause progressive myoclonus epilepsy^116^ or an epilepsy syndrome associated with ataxia^117^ called myoclonus epilepsy and ataxia due to potassium channel mutation (MEAK), whereas mutations in K_V_3.3, a subunit that is highly expressed in cerebellar Purkinje cells, cause spinocerebellar ataxia type 13 (SCA13).^118^ Together, these studies provide target validation in humans suggesting that K_V_3 channel activators could be useful for treating excitability disorders such as epilepsy and ataxia and potentially also psychiatric disorders including schizophrenia.^119^

#### 1 K_V_3.1/3.2 potentiators have therapeutic potential for Fragile X, genetic epilepsy, and schizophrenia

Increasing K_V_3 channel activity with small molecular modulators that lower their activation voltage could restore a disturbed excitation-inhibition balance by, for example, increasing the firing rate of fast-spiking GABAergic interneurons in the hippocampus.^120^ This therapeutic hypothesis was pursued by Autifony Therapeutics, a company that discovered several K_V_3 channel potentiating tool compounds and drug candidates including the imidazolidinedione-based K_V_3.1/3.2 channel modulator AUT1.^121^ The cryo-EM structure of K_V_3.1 bound to the more potent derivative AUT5, showed that 4 AUT5 molecules bind in the interface between the membrane-spanning S4 segments from the voltage-sensor domains and the loops on top of the S5 segment of the neighboring pore domains (PDs), where ligand binding induces quite a substantial conformational change.^122^ Another dual K_V_3.1/3.2 potentiator was recently reported from a schizophrenia drug discovery program at Merck.^123^ The compound has submicromolar activity and 4 molecules were found to bind in the same extracellular pocket between the turret and the voltage-sensor domain by presumably displacing cholesterol which might occupy the space in the apo state.^123^ Both these compounds thus differ in their binding mode from another class of K_V_3 channel-positive gating modulators exemplified by AG00563 from Lundbeck where the cryo-EM structure showed 1 ligand occupying a site further down in the interface between PD and voltage-sensor domain on the intracellular side of the channel.^124^

Although Lundbeck and Merck do not seem to have moved their K_V_3.1/3.2 potentiators into the clinic, Autifony sponsored a phase IIa study for tinnitus in England for AUT00063 in 2019, which failed to show alleviation of tinnitus symptoms but demonstrated that the drug achieved the desired plasma levels and was safe and well tolerated.^125^ The company recently completed a phase Ib trial (NCT05873062) with the K_V_3.1/3.2 activator AUT00201 in patients with the orphan epilepsy syndrome MEAK, with results available in ClinicalTrials.gov. Another K_V_3.x channel activator, AUT00206, seems to be slated to begin phase II clinical trials for Fragile X syndrome, according to the company website, following efficacy studies in fmr1 knockout mice, a model of Fragile X, demonstrating improved cognitive function and reduced behavioral abnormalities. AUT00206 has also been tested in phase Ib studies in patients with schizophrenia. Although these studies so far only demonstrated pharmacodynamic effects of K_V_3 channel activation in individual patients,^126^ it is possible that subgroups of patients in larger trials might respond more robustly to this nondopaminergic treatment and more thorough investigations in larger trials are certainly warranted.^127^ The underlying therapeutic hypothesis that modulation of K_V_3.1/3.2 channels in the basal ganglia could reestablish inhibitory GABA control and thereby inhibit overactive dopamine neurons and restore the balance of excitation and inhibition in cortical circuits in schizophrenia^128^ is certainly attractive.

### C K_V_7 channel activation to reduce neuronal excitability in amyotrophic lateral sclerosis, epilepsy, and major depression

The K_V_7.x or *KCNQ* family channels are characterized by a low activation threshold voltage (∼60 mV), slow kinetics, and little inactivation, properties that are ideally suited for controlling excitability.^129^ K_V_7.1 is expressed in the vasculature and in the heart, where it underlies the slow delayed rectifier current I_Ks_.^130^^,^^131^ K_V_7.1 inhibition was pursued as an antiarrhythmic approach in the late 1990s and early 2000s, but none of the compounds developed by Merck^132^ or Sanofi-Aventis^133^^,^^134^ seems to have moved beyond studies in dogs or pigs at that time.^11^ The other 4 channels, K_V_7.2–7.5 (*KCNQ2-5*), are widely expressed in the CNS.^130^^,^^135^ Heteromultimers predominantly consisting of K_V_7.2 and K_V_7.3 carry the so-called “M current,” named after its muscarinic modulation, and function as breaks on neuronal firing.^129^ LOF mutations in *KCNQ2* or *KCNQ3* are associated with various forms of epilepsy in humans ranging from benign familial neonatal epilepsy to severe EE with cognitive impairment or pharmacoresistant seizures such as neonatal EE.^136^, ^137^, ^138^, ^139^ A recent review article provides an excellent and extensive summary of the K_V_7 channel modulator pharmacology and the efforts of many pharmaceutical companies to target K_V_7 channel subtypes with both inhibitors and activators for a large number of therapeutic indications during the last 3 decades.^140^ K_V_7 channels also seem to be affected by many botanical folk medicines.^141^

#### 1 Beyond retigabine: Novel positive allosteric modulators of K_V_7.2/7.3 channels

In keeping with the crucial role of K_V_7 channels in regulating neuronal excitability, K_V_7.2/7.3 channel openers, which should correctly be called PAMs or activators, because they typically act by shifting the half-activation voltage of these channels to more negative voltages, have long been pursued as antiseizure drugs.^142^ The relatively nonselective K_V_7 channel PAM retigabine was FDA-approved as ezogabine in 2011 for the treatment of partial seizures but discontinued in 2017 due to side effects including urinary retention, retinal abnormalities, skin discolorations, and limited use.^142^

After some attempts at repurposing of retigabine for ALS in a phase II clinic trial (NCT02450552), where it was shown to reduce cortical and spinal motor neuron hyperexcitability in ALS patients using transcranial magnetic stimulation,^143^ or reformulation as XEN496 for *KCNQ2*-related DEE and EE,^142^ there are currently multiple new K_V_7.2/7.3 PAMs in various stages of clinical trials. QurAlis is performing phase I clinical studies with its compound QRL-101 for both ALS and potential antiseizure effects using electroencephalogram brain activity as a biomarker for target engagement similar to the published retigabine study in ALS.^143^ The therapeutic hypothesis for ALS is that K_V_7 channel activation could reduce hyperexcitability-induced motor neuron degeneration.^143^ Another company developing a new K_V_7 PAM is Xenon Pharmaceuticals, whose Azetukalner (a.k.a. encukalner or XEN1101) demonstrated positive phase IIb data in adults with focal epilepsy^144^ and is now in a phase III study for the treatment of focal onset seizures (NCT05716100). Xenon is also advancing XEN1101 for the treatment of major depressive disorder and after a phase II study (NCT05376150) that showed improvements at the higher dose, just started recruiting for a phase III study with 450 patients for moderate-to-severe major depressive disorder (NCT06775379). A follow-on compound, XEN1120, just started phase I safety studies and could be developed as an analgesic.

Similarly, Biohaven is advancing BHV-7000 (opakalim), a K_V_7.2/7.3 PAM in phase II trials for bipolar disorder (NCT06419608) and in several ongoing phase III studies for generalized (NCT06425159) and focal epilepsy. However, the bipolar disorder trial recently failed to meet is primary endpoint in reducing the Young Mania Rating Scale, but the results are being further analyzed and have not been posted in ClinicalTrials.gov. Another, apparently highly K_V_7.2/7.3 selective PAM that is just starting clinical development for epilepsy is SAN2355 from the Danish company Saniona.

### 2 K_V_7.4 channel activation for hearing loss

Another interesting clinical trial in the K_V_7-field is a phase IIa study (NCT06521190) from the German company Acousia Therapeutics that is testing the efficacy of the K_V_7.4 activator ACOU085 (bifocaled) administered into 1 middle ear of patients prior to chemotherapeutic cycles with cisplatin, which induces hearing loss. The contralateral ear of each patient will serve as intraindividual placebo-injected control. The therapeutic premise behind this trial is that K_V_7.4 channels mediate the predominant K^+^ conductance of auditory outer hair cells and that *KCNQ4* (K_V_7.4) LOF mutations can cause hereditary hearing loss.^145^ Channel activation with retigabine has been shown to rescue function.^145^^,^^146^

## V Cryo-electron microscopy reveals the molecular mechanisms of voltage-gated ion channel-targeting drugs, guiding next-generation therapeutics

A detailed structural understanding of VGIC function and modulation at the atomic level could enable structure-based drug design. Foundational X-ray studies in the early 2000s revealed the first structure of a voltage-sensor in the bacterial K_V_ channel K_V_AP^147^ and elucidated the molecular mechanisms of electromechanical coupling between voltage-sensor and pore via the S4–S5 linker in the mammalian K_V_1.2 structures.^148^^,^^149^ Although these foundational studies provided critical structural details, most VGICs remained structurally elusive for years, as their complexity posed significant challenges for X-ray crystallography. However, over the past decade, the cryo-EM revolution has provided the field of ion channel pharmacology with many high-resolution cryo-EM structures of VGICs, both in their apo states and bound to a wide array of modulators. This structural wave, driven by advances in electron microscope hardware, 3D-image classification, and membrane protein structural biology, has begun to elucidate how clinically used drugs and investigational compounds interact with their VGIC targets. What had previously only been suspected, namely that many drugs bind in a state-dependent manner, is now visible at the atomistic level with cryo-EM structures showing drugs bond to the open, closed, or inactivated conformation of channels.^9^^,^^23^^,^^150^ These developments are starting to enable the next-generation of rational drug design efforts grounded in structure-based principles rather than empirical screening alone. Pivotal to this progress has been the development of deep expertise for the purification and structural determination of specific ion channel subfamilies, initially by academic laboratories. These efforts have resulted in a growing repository of structural data that elucidates the detailed mechanisms of VGIC modulation and offers insights into how pathogenic mutations affect channel function.

In the sodium channel field, the crystal structure of the bacterial Na_V_Ab channel revealed the basic architecture of the pore and selectivity filter, as well as lateral pore fenestrations implicated in drug access through lipid environment in 2011.^151^ Although prokaryotic Na_V_ channels continued to be resolved, eukaryotic Na_V_ channels remained elusive for structural determination due to their large size. A major leap forward came in 2017 with the cryo-EM structures of the first eukaryotic Na_V_ channels, Na_V_PaS from the American cockroach,^152^ and Na_V_1.4 in complex with the *β*1 subunit from the electric eel,^153^ followed in 2018 by its human ortholog,^154^ which displayed the fast inactivation gate, the IFM motif, docked into a hydrophobic receptor formed by the domain III–IV interface, validating decades of functional data.^7^^,^^155^, ^156^, ^157^ A 2019 structure of a human-cockroach hybrid Na_V_ channel bound to an *α*-scorpion toxin illuminated the allosteric mechanisms of fast inactivation.^158^ Subsequent studies have significantly broadened this structural repertoire, with high-resolution experimental structures now available for all human Na_V_ subtypes except Na_V_1.9. These include complexes with diverse small molecules and peptide toxins,^159^, ^160^, ^161^, ^162^ revealing their modulatory mechanisms and selectivity determinants. Structural characterization of clinically relevant variants, such as the Na_V_1.5-E1784K mutant associated with both Long QT syndrome type 3 and Brugada syndrome,^163^ has begun to provide insights into the molecular basis of how gating perturbations arise from single residue changes. Most recently, a 2024 structure captured a distinct slow-inactivated state of Na_V_Eh featuring a dilated selectivity filter.^164^

For Ca_V_ channels, a major milestone came in 2015 with the cryo-EM structure of the rabbit skeletal muscle Ca_V_1.1 complex, which revealed how the *α* subunit assembles with its auxiliary *β*, *α*2*δ*, and *γ* subunits.^165^ In 2019, structures of Ca_V_1.1 bound to the calcium channel blockers nifedipine, verapamil, and diltiazem revealed distinct drug-binding pockets and entry pathways.^166^ That same year, the first structure of a human low-voltage-activated T-type channel, Ca_V_3.1, was solved in both apo form and in complex with the selective inhibitor Z944.^71^ In the years that followed, structural coverage of members of the different Ca_V_ subfamilies expanded steadily. A 2021 study resolved the human Ca_V_2.2 channel bound to ziconotide, a pore-blocking peptide toxin approved for chronic pain treatment.^167^ In 2022, structures of the human Ca_V_1.3 channel in apo form and in complex with cinnarizine were published,^168^ alongside the first apo structure of human Ca_V_2.3^169^ and the apo and modulator-bound structures of Ca_V_3.3.^170^ The structure of human Ca_V_1.2 appeared in 2023,^171^ alone and in complex with multiple drugs and the peptide toxin calciseptine. Most recently, in 2024, structures of human Ca_V_1.2 bound to tetrandrine and benidipine,^172^ human Ca_V_3.2 in its apo state and in complex with selective antagonists,^173^ and the human Ca_V_2.1 channel in both apo form and bound to peptide toxins were reported.^174^ In addition to resolved Ca_V_ assemblies, a recent structure of human Ca_V_1.2 in complex with the endoplasmic reticulum membrane protein complex chaperone has provided access to assembly intermediates.^175^ This study revealed how the endoplasmic reticulum membrane protein complex transiently engages Ca_V_1.2 during biogenesis and highlighted mutually exclusive interactions with Ca_V_*α*2*δ*, suggesting a stepwise, chaperone-guided assembly pathway. These findings offer a window into the in vivo assembly process of Ca_V_ channels and could provide new strategies for modulating channel function by targeting specific steps in their biosynthetic pathway.

Parallel advancements in the K_V_ channels field, including breakthrough structures of K_V_11.1 (hERG) and K_V_7.1 (*KCNQ1*) resolved between 2017 and 2020,^176^^,^^177^ have provided insights into K_V_ channel inactivation mechanisms, calmodulin modulation, and structural determinants of arrhythmogenic effects. In 2021, structures of K_V_7.2 (*KCNQ2*) and K_V_7.4 (*KCNQ4*) revealing the molecular mechanisms of both activators and inhibitors were resolved.^178^^,^^179^ More recently, the 2022 structure of human K_V_3.1 uncovered a gating mechanism mediated by the cytoplasmic T1 domain,^180^ offering a structural basis for the fast kinetics of these channels. Newly resolved conformations of K_V_1.2 and K_V_1.3 revealed different selectivity filter states and hydrogen bond networks, providing mechanistic insight into the possible structural determinants of C-type inactivation.^181^, ^182^, ^183^ In parallel, cryo-EM structures of K_V_2.1 from 2023, including variants linked to EEs, highlighted a conserved hydrophobic coupling nexus near the intracellular pore end that modulates slow inactivation through electromechanical coupling.^184^ A novel strategy to solve ion channel structures in lipid membrane vesicles with an applied electric field by using K^+^ ion gradients in the presence of valinomycin was reported in 2022.^185^ When applied to K_V_7.1 (*KCNQ1*), this method identified a novel indirect gating mechanism for K_V_7.1 channels in which voltage-sensor movements modulate PIP_2_ accessibility and binding, which ultimately triggers pore opening, rather than exerting direct mechanical force on the pore.^186^ Most recently, a 2024 high-resolution structure of K_V_3.1 in complex with AUT5, a selective PAM, revealed an extracellular binding pocket at the interface of the voltage-sensing and PDs.^122^ Allosteric engagement at this site stabilizes the open state through turret rearrangement, offering a structural foundation for therapeutic K_V_3 activity enhancement in hyperexcitability isorders.^122^

As experimental protocols for VGIC structure determination have become increasingly optimized and disseminated across the academic community, pharmaceutical companies have begun to implement cryo-EM as a cornerstone of early-stage drug discovery. Several companies established in-house structural biology platforms dedicated to high-throughput determination of channel drug complexes for priority targets. For example, Genentech obtained several structures to guide the optimization of their Na_V_1.7 selective aryl-sulfonamides,^27^ whereas investigators at Merck more recently reported K_V_3.1 structures from a K_V_3.1/3.2 potentiator drug discovery program for schizophrenia.^123^ Other companies are turning to contract research organizations such as NanoImaging Services, which now offers cryo-EM–based structural determination services, including the resolution of drug-bound complexes. After publicly reporting the successful determination of a 2.7 Å structure of the hERG (K_V_11.1) potassium channel, the canonical off-target in cardiac safety, in complex with the antihistamine astemizole,^187^ NanoImaging Services is now offering this capability as a consultative service for clients seeking to evaluate hERG liability at atomic resolution.

In the next section, we will discuss the binding sites revealed by these structural studies and examine the molecular mechanisms through which both classical and investigational compounds modulate VGIC activity. For a comprehensive and up-to-date review of the structural pharmacology of VGICs and a proposal for naming druggable sites, we refer the reader to a recent outstanding review by Huang et al.^9^

### A Binding sites for voltage-gated Na^+^ channel modulation

**Fig. 3:**
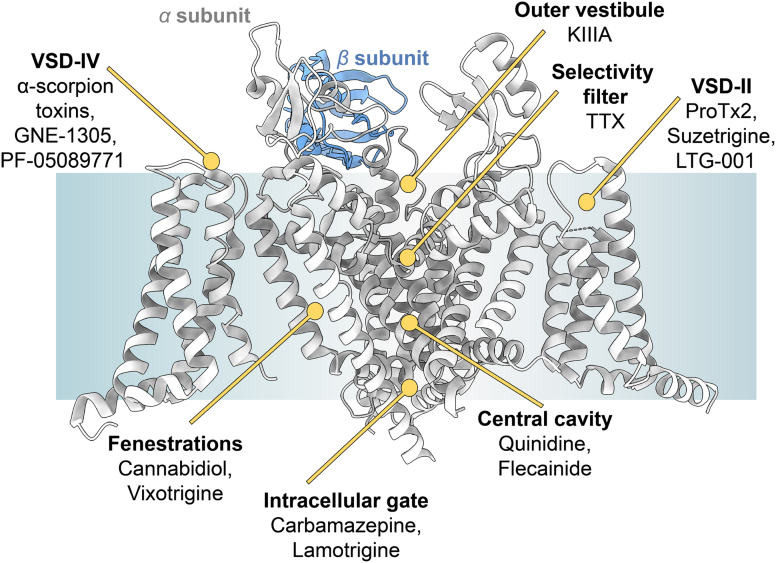
Binding sites of known modulators of Na_V_ channels. General structural model of a Na_V_ channel highlights key drug-binding sites on the *α* subunit (gray); *β*2 is shown for reference. Based on the experimental structure Protein Data Bank: 7W9K. Figure generated with ChimeraX.

Molecules targeting Na_V_ channels have historically been categorized as either pore blockers or gating modifiers based on functional studies.^7^ However, with the recent availability of high-resolution experimental structures, ligand binding sites can now be visualized at the atomistic level (Fig. 3). Broadly, Na_V_ channel modulators engage either the PD or the voltage-sensing domains (VSDs), with VSD-targeting compounds exerting their effects by altering gating behavior in a state- and domain-specific manner. For instance, modulators such as ProTx2 and suzetrigine stabilize the channel in a closed state by binding to VSD-II in its deactivated conformation.^2^^,^^160^ Ligands that interact with voltage-sensing domain IV (VSD-IV) influence fast inactivation, *α*-scorpion toxins, by stabilizing VSD-IV in a deactivated state, inhibit fast inactivation, and induce sustained sodium currents^158^; conversely, compounds like GNE-1305 and PF-05089771 bind to an activated conformation of VSD-IV, hindering recovery from inactivation and thereby stabilizing inactivated, nonconductive channels.^27^ Notably, VSD-binding molecules exhibit diverse binding modes: some interact exclusively with extracellular VSD loop regions (eg, ProTx2), others engage both the VSD and adjacent PD (as with *α*-scorpion toxins), whereas a third class inserts directly into the central cavity of the VSD, establishing interactions with transmembrane residues (such as GNE-1305). The PD has a series of distinct binding pockets (Fig. 3), spanning from the outer vestibule, where conotoxin KIIIA binds to the selectivity filter targeted by TTX, the central cavity, where classical antiarrhythmic drugs such as quinidine and flecainide bind to the intracellular activation gate engaged by compounds like carbamazepine and lamotrigine.^9^ Moreover, membrane-facing fenestrations within the PD provide alternative binding sites for hydrophobic ligands such as cannabidiol and vixotrigine,^188^ which can partition directly from the lipid bilayer into their respective binding pockets. Importantly, many pore-targeting compounds bind within regions whose structural and physicochemical properties vary with the channel’s conformational state, allowing these drugs to modulate channel gating. Together, these observations highlight the extraordinary adaptability of Na_V_ channels to accommodate a wide range of chemical entities across structurally and functionally distinct regions. Wherever the structure permits, molecules can engage the channel with modulatory consequences even in shallow or transient pockets. Due to this complexity, a new detailed nomenclature has recently been proposed to classify Na_V_ binding sites with greater precision and consistency.^150^

### B Binding sites for voltage-gated Ca^2+^ channel modulation

**Fig. 4:**
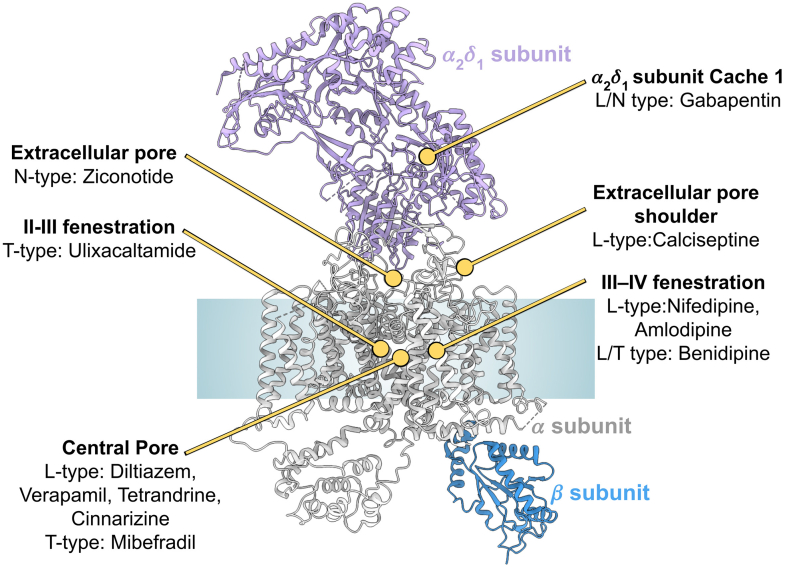
Binding sites of known modulators of Ca_V_ channels. General structural model of a Ca_V_ channel highlights key drug-binding sites on the *α* (gray), *β* (blue), and *α*_2_*δ*_1_ (light purple) subunits. Based on the experimental structure Protein Data Bank: 8FD7. Figure generated with ChimeraX.

Small molecule ligands targeting Ca_V_ channels engage a range of structurally distinct binding pockets (Fig. 4). Among the most extensively characterized are the 1,4-DHPs, such as nifedipine and amlodipine, which bind laterally in the repeat III–IV fenestration of the PD, as captured in high-resolution structures of rabbit Ca_V_1.1.^189^ Notably, amlodipine distinguishes itself by engaging not only this classical fenestration site but also occupying a second binding pocket within the central cavity, overlapping with regions targeted by non-DHP pore blockers, such as verapamil and diltiazem. This dual binding mechanism likely explains amlodipine’s high potency and prolonged duration of action.^166^ The central cavity, accessible via the II–III fenestration, accommodates ligands such as verapamil and diltiazem in the Ca_V_1.1^71^ and mibefradil in Ca_V_3.3.^170^ Ulixacaltamide (Z944), a T-type-selective antagonist, exploits this evolutionarily conserved access pathway but binds directly within the II–III fenestration of Ca_V_3.1 (Fig. 4), obstructing ion flow through lateral pore occlusion rather than deep central cavity engagement.^71^ Beyond the pore-forming *α*1 subunit, Ca_V_ channels are modulated by ligands targeting auxiliary components. Gabapentin binds to the extracellular *α*2*δ*-1 subunit (Fig. 4), specifically within a deep pocket of its dCache1 domain. Cryo-EM studies indicate minimal structural differences between gabapentin-bound and apo states of *α*2*δ*-1, suggesting that gabapentin’s mechanism of action may involve dynamic or indirect modulation rather than conformational locking.^190^

Peptide toxins, in contrast, act via surface-accessible interfaces (Fig. 4). Ziconotide, a synthetic *ω*-conotoxin MVIIA, blocks Ca_V_2.2 by physically occluding the outer pore entrance, displacing the extracellular loop of repeat III and the *α*2*δ*-1 subunit to prevent ion permeation.^167^ Calciseptine, a snake venom toxin, binds peripherally on the channel’s outer “shoulder,” contacting repeats III and IV.^171^ Although distal from the pore, this site may allosterically stabilize the closed state to inhibit conductance.

### C Binding sites for voltage-gated K^+^ channel modulation

**Fig. 5:**
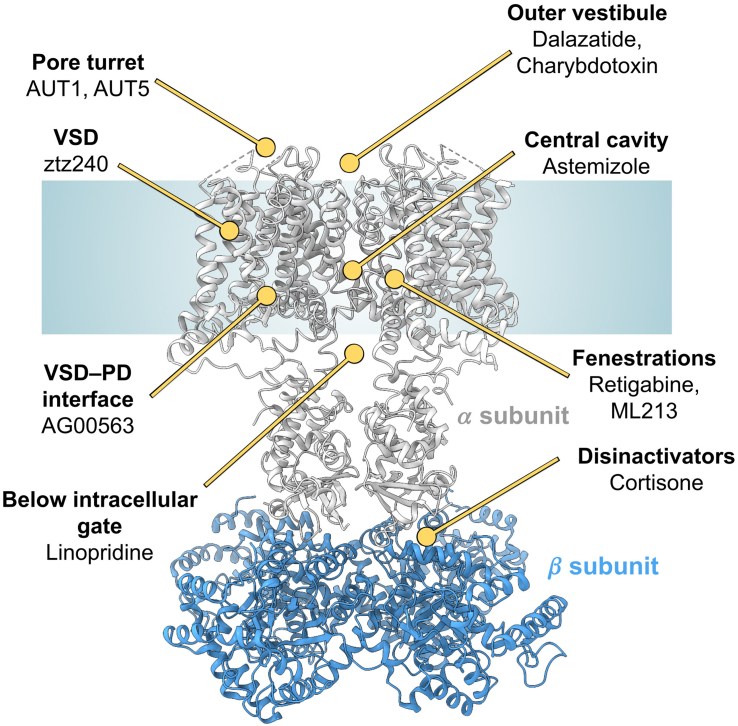
Binding sites of known modulators of K_V_ channels. General structural model of a K_V_ channel highlights key drug-binding sites on the *α* subunit (gray) and associated auxiliary *β* subunits. Based on the experimental structure Protein Data Bank: 7EJ1. Figure generated with ChimeraX.

Overall, K_V_ channels offer similar binding sites for small molecules and peptides as Na_V_ and Ca_V_ channels (Fig. 5). However, in contrast to Na_V_ and Ca_V_ channels, which often are only pseudo-symmetric because of sequence differences in the 4 repeats of the single polypeptide chain forming the channel, K_V_ channels are tetramers composed of 4 individual subunits and are fully symmetric when they are homotetramers, meaning they consist of 4 identical *α* subunits.^9^^,^^16^ Because of the challenges associated with working with recombinantly expressed heteromultimers, so far, all structural studies of K_V_ channels have been performed with homotetramers and not with heteromultimers, which can differ in their biophysical and pharmacological properties.^16^

Binding sites for K_V_ channel modulators have been mapped to multiple regions within the PD (Fig. 5). Some ligands, such as the pore-blocking peptide toxins charybdotoxin,^191^ the ShK-derivative dalazatide,^192^ and an ShK-antibody fusion protein,^182^ bind to the extracellular vestibule directly above the selectivity filter, physically occluding the ion conduction pathway with a positively charged lysine residue. Others, like the K_V_11.1 (hERG) inhibitor astemizole occupy the central cavity,^187^ whereas the K_V_7 channel blocker linopirdine has been captured in K_V_7.4 below the intracellular gate.^179^ Fenestrations have also been revealed as druggable sites in K_V_ channels, as structural studies of K_V_7.2 and K_V_7.4 in complex with the channel openers retigabine and ML213 revealed ligand binding within these lateral pockets.^178^^,^^179^^,^^193^

The K_V_3.1 PAM AG00563 binds at the interface between the VSD and the PD on the intracellular side of the membrane to stabilize the open state.^124^ Within the VSD cleft of K_V_7.2, the small molecule potentiator ztz240 was found to bind and stabilize the activated “up” conformation of the S4 helix, effectively promoting channel opening.^178^ More recently, a distinct extracellular binding site (Fig. 5) was discovered at the inter-subunit interface between the VSD and the PD in K_V_3 channels.^122^ Here, AUT5 acts as a PAM by promoting turret rearrangements that establish new interactions with the VSD, effectively immobilizing the S4 segment in its activated position. Interestingly, for both AUT5 and another K_V_3 channel potentiator 4 molecules were observed in the structure occupying all 4 extracellular pockets around the turret.^122^^,^^123^ Similarly, for a K_V_1.3 blocking nanobody, 4 nanobodies were seen to “decorate” the 4 turret loops and bridge them to the VSDs with 4-fold symmetry.^182^ Finally, targeting auxiliary subunits offers another strategy for modulation: cortisone, a steroid hormone, has been shown to disrupt the interaction between K_V_*β* and its associated *α*-subunit.^194^ This mechanism prevents auxiliary subunit-dependent inactivation and thereby potentiates channel activity, a mode of action for which the compound has been termed a “disinactivator.”

## VI Strategies integrating structural data, human insight, and deep learning in voltage-gated ion channel drug discovery

**Fig. 6:**
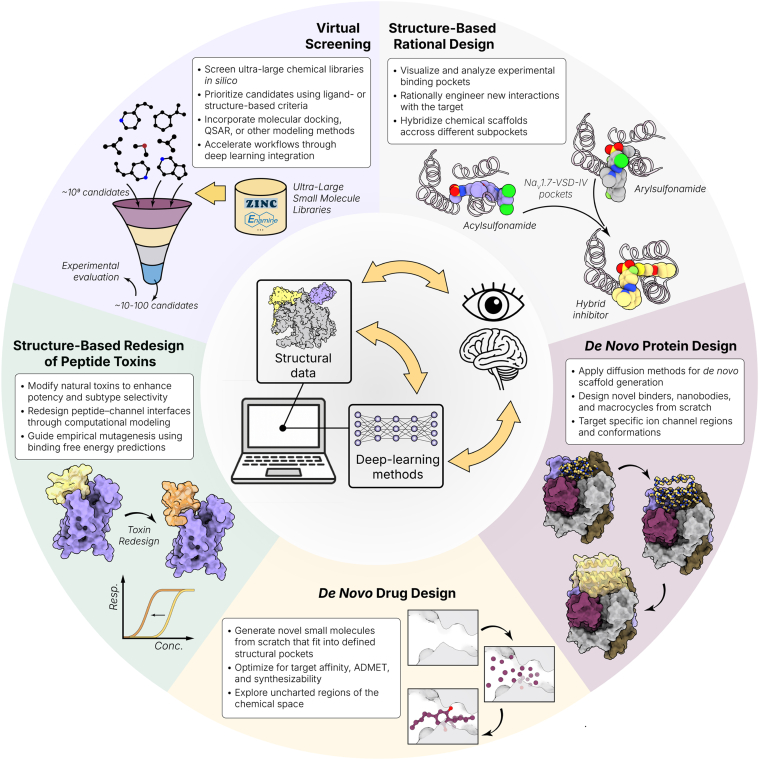
Outlook on computational strategies using structural data, VS and deep learning in VGIC drug discovery. Newly emerging approaches harness structural data and AI, integrating human reasoning to guide the rational design of VGIC-targeted modulators. Shown here are 5 representative strategies. See the main text for details and examples applied to VGIC targets. Structural models generated with ChimeraX. ADMET, absorption, distribution, metabolism, elimination, toxicity; QSAR, quantitative structure-activity relationship.

Current computational methods enable the in-depth analysis of experimental structures with relatively low requirements for computational skills. With training, users can visualize binding pockets, extract interaction motifs, estimate energetic contributions, and generate structure-informed hypotheses that feed directly into the design cycle. Although such capabilities have long been available and widely used for soluble targets like enzymes with several clinically approved kinase inhibitors resulting from such approaches,^195^^,^^196^ their application to VGICs has historically been limited by the lack of high-resolution structural data. The recent wave of cryo-EM structures allows these same structure-guided strategies to be applied to VGICs. Moreover, in parallel to the increasing the availability of structures, computational drug discovery methods have also begun to adapt deep learning (Fig. 6), that is, AI models based on artificial neural networks. VS campaigns, predictive binding affinity assessments, and even the de novo engineering of functional molecules such as nanobodies or entirely novel scaffolds are incorporating deep learning for increased throughput.^197^, ^198^, ^199^ However, when applied to VGIC, where conformational complexity and membrane embedding pose unique challenges, many of these deep learning applications remain at the proof-of-concept stage. Nonetheless, in the following sections, we outline the range of projects made possible by these advancements and highlight early efforts to apply them to VGIC targets.

### A Rational structure-based design for voltage-gated ion channels

Besides understanding how a drug molecule interacts with its target, experimental structures can generate hypotheses and drug design strategies by making it possible to deconstruct pharmacophores, engineer new interactions, and even hybridize distinct chemical scaffolds into larger molecules that engage with multiple subpockets within the same target. This capacity to iteratively “see and reason” at the molecular scale finally enables structure-based drug design for VGICs. A compelling illustration of this strategy is provided by the above-mentioned study at Genentech, where a multidisciplinary team used cryo-EM to resolve 2 chemically distinct classes of Na_V_1.7-selective inhibitors, arylsulfonamides^27^ and acylsulfonamides.^43^^,^^200^ Despite targeting the same VSD-IV region, the 2 compound classes were found to engage distinct pockets, 1 between S2 and S3, the other between S3 and S4.^201^ This unexpected difference in binding sites changed the prevailing assumption that both chemical classes acted through a common mechanism and instead suggested that Na_V_1.7’s VSD-IV was flexible and capable of accommodating ligands with different chemical topologies and entry pathways into its binding pocket. This insight motivated a bold design hypothesis: if a single molecule could span the aryl- and acyl-binding pockets, 1 might develop a hybrid inhibitor capable of synergistically engaging both domains while reducing dependence on lipophilic, membrane-anchoring tail groups (Fig. 6). Indeed, the resulting compounds, including GNE-9296 and GNE-1305, validated this strategy.^201^ Cryo-EM structures revealed that these hybrid inhibitors establish stabilizing interactions across both binding clefts, allowing them to simultaneously access selectivity determinants from the S2/S3 and S3/S4 pockets. Of particular note, the GNE-1305 structure exhibited efficient space-filling without relying on lipid-facing extensions,^201^ a structural modification that improved pharmacokinetic properties. This work demonstrates the power of structural insights.

### B Navigating ultra-large chemical libraries with deep learning–accelerated virtual screening

**Fig. 7:**
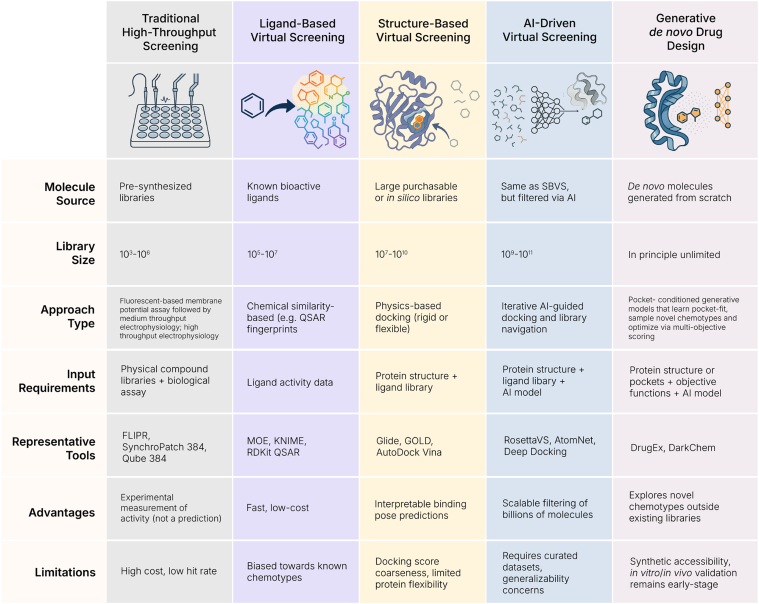
Comparison of screening and design strategies for small molecule modulators of VGICs. Each method varies in input requirements, scalability, and capacity to explore chemical space, offering distinct advantages and limitations. QSAR, quantitative structure-activity relationship.

VS refers to the rapid, computational evaluation of large, virtual, small molecule libraries,^202^ to identify candidate molecules with predicted affinity for a biological target. VS assists and extends traditional high-throughput screening experimental approaches by guiding prioritization of compounds to be tested, allowing for the exploration of larger libraries (Fig. 7). For a comprehensive review of the state-of-the-art application of VS to ion channel targets, we refer readers to the following publication.^203^ Although the extent to which the combination of structural data availability, ultra-large chemical libraries and deep learning will reduce reliance on experimental high-throughput screening remains to be seen, there is little doubt that these techniques have the potential to accelerate early-stage discovery.

#### 1 Chemical libraries for virtual screening

The scalability of VS has expanded dramatically in recent years. Early-generation libraries such as ZINC held just over 7000 compounds in 2005; by 2025, the same platform offers free virtual access to over 2 billion purchasable molecules.^204^ Enamine’s readily accessible (REAL) Space library,^205^ now exceeding 64.9 billion compounds, and enables VS across an enormous chemical space, with the option to “cherry-pick” and order only the most promising candidates for on-demand synthesis and subsequent experimental testing. Smaller, target-focused libraries^206^ are also available, such as ChemDiv’s or Enamine’s ion channel-targeted libraries containing 26,000 or 40,000 small molecules curated for major ion channel families. This explosion in accessible chemical space for VS has made it necessary to develop computational tools capable of navigating these immense chemical libraries.

VS methods (Fig. 7) generally fall into 2 categories^207^: ligand-based VS and structure-based VS (SBVS). Ligand-based VS relies on existing ligands to identify new hits with similar properties, using tools such as quantitative structure-activity relationship models. In contrast, SBVS uses the 3D structure of a protein target to predict how small molecules bind and predict binding affinities, often through molecular docking approaches. SBVS is particularly relevant for VGICs now that high-resolution cryo-EM structures have become available. Docking programs such as Cambridge Crystallographic Data Centre Gold,^208^ Glide,^209^ and Autodock Vina^210^ can evaluate the conformational “fit,” meaning the shape complementarity of small molecules in defined binding pockets, treating the protein either as a rigid body or allowing limited conformational flexibility, such as side chain rotations, in response to ligand binding and predict binding affinities. However, scoring functions remain a challenge for membrane-embedded systems like VGICs where electrostatics and lipid interactions are critical. Recent developments have led to more sophisticated, context-specific scoring functions; for instance, Rosetta,^211^^,^^212^ HADDOCK,^213^ and LightDock^214^ offer scoring frameworks in implicit membrane models. The Schrödinger suite of computational tools enables the use of Desmond for membrane-embedded MD, with results that can be transferred to Glide for docking. Although MD provides more accurate binding predictions, it is computationally intensive and time consuming, motivating the use of alternative methodologies such as free energy perturbation methods.^215^ Several successful applications of SBVS to ion channel targets have been reported. For example, docking campaigns targeting the VSD of the K_V_7.1 channel identified novel activators by exploiting pockets specific to conformational states.^216^ Studies on other ion channels reported successful identification of compounds targeting TRPV5,^217^ inward-rectifier Kir channels,^218^ K_Ca_1.1 (BK) channels,^219^ and nicotinic acetylcholine receptors^220^ from in silico screening.

#### 2 Integration of artificial intelligence into virtual screening for voltage-gated ion channels

The increasing size of libraries described above has led to the incorporation of AI in VS workflows (Fig. 6). Deep learning-accelerated platforms such as Deep Docking,^221^ RosettaVS,^222^ and AtomNet^223^ represent a new generation of computational screening tools that combine physics-based modeling with AI to improve both accuracy and speed (Fig. 7). The deep docking platform exemplifies this synergy by enabling up to 100-fold acceleration of traditional SBVS workflows and resulted in 6000-fold enrichment of high-scoring molecules in published benchmarking. Deep docking docks only a subset of a large chemical library while it iteratively trains a ligand-based quantitative structure-activity relationship model to predict docking scores for the remaining molecules, thus prioritizing which compounds are worth evaluating next.^221^ RosettaVS represents another integrated approach, that explicitly models receptor flexibility and trains a target-specific feed-forward network to prioritize compounds to dock in subsequent iterations.^222^ In proof-of-concept studies, RosettaVS was applied to the Enamine-REAL (∼5.5 billion compounds) or the ZINC22 library (∼4.1 billion compounds) for targeting the E3 ubiquitin ligase substrate receptor (KLHDC2) and the human Na_V_1.7 channel.^222^ For Na_V_1.7, 4 distinct hits with single-digit micromolar affinity were experimentally confirmed, yielding a 44% hit rate.^222^

Atomwise also pioneered deep learning-based VS through its AtomNet platform and the recently introduced AtomNet PoseRanker.^223^ This method, a graph convolutional network, learns to identify high-quality ligand poses from sampled ensembles of target conformations. In the largest VS campaign reported to date,^224^ encompassing 318 drug discovery projects across all major protein families including GPCRs, kinases, proteases, and ion channels, AtomNet identified novel hit compounds even for targets without previously known binders or high-quality experimental structures. Notably, ion channels accounted for 4% of the targets screened, with successful hits reported for HCN2 (hit rate 5.45%), Ca_V_1.2 (hit rate 2.63%), and the calcium-activated K_Ca_3.1 channel (hit rate 3.61%).

### C Generative drug design enables access to unexplored chemical space

Although still in its early stages, generative drug design, also known as de novo drug design (Fig. 7), is rapidly emerging as a powerful complement to traditional VS methods.^225^ These approaches do not search in libraries of existing or virtual molecules but instead aim to create novel chemical entities from scratch that “fit” into given binding pocket targets (Fig. 6). By “learning” the binding patterns of bioactive molecules in complex with their protein targets, as well as their associated physicochemical properties, generative models can generate new molecules predicted to display favorable binding affinity, drug-likeness, pharmacokinetic profiles, and synthetic accessibility,^225^ opening routes into unexplored regions of chemical space. Recent proof-of-concept studies have begun to explore the applicability of these techniques to ion channel targets. For example, the DarkChem platform^226^ has been used to generate candidate antagonists for the phencyclidine site of the *N*-methyl-*D*-aspartate receptor.^227^ DrugEx successfully yielded ligands optimized for binding to adenosine receptors while simultaneously avoiding off-target interactions with hERG channels.^228^

### D Computational structure-based redesign of peptide toxins

Peptide toxins from the venoms of scorpions, spiders, sea anemones, and cone snails potently inhibit VGICs through various mechanisms primarily targeting the outer pore or VSDs in a resting or activated conformation.^229^ The majority of these peptides are distinguished by a combination of disulfide bonds forming a “knot-like” secondary structure to stabilize the peptide fold: an inhibitor cystine knot motif.^230^ Developing biologic derivatives from peptide toxins remains an attractive modality due to their high potency, and lack of drug-drug interactions exhibited by small molecules.^6^^,^^231^ However, an often-encountered challenge is designing subtype-selective peptides, which overcome the narrow therapeutic safety window often found with venom-derived peptides, since these peptides are utilized to paralyze prey. There are numerous reviews discussing the therapeutic potential and challenges of peptide toxins targeting VGICs.^229^^,^^231^, ^232^, ^233^

We will here describe efforts utilizing Protoxin-II (ProTx2) as a template for the design of an inhibitor of the pain target hNa_V_1.7 to illustrate the prior, current, and future use of peptides as a modality, as well as exemplify the use of computational protein redesign to guide the optimization and screening of peptide toxin variants. ProTx2 was identified more than 15 years ago by scientists at Merck to selectively target hNa_V_1.7 (0.3–1 nM IC_50_ and 30–100-fold hNa_V_1.7 selectivity) and block action potential propagation responsible for nociception.^234^ Prior efforts to optimize ProTx2 for hNa_V_1.7 were structurally blind screening programs creating and testing large compound libraries of peptide toxin mutants.^235^ Preclinical development of ProTx2 variants was spearheaded by Janssen Biotech, which demonstrated that intrathecal injection of ProTx2 in rats exerted a strong analgesic effect; however, ProTx2 had a narrow therapeutic window and inhibited other Na_V_ subtypes (Na_V_1.1/Na_V_1.6) at moderately higher doses, resulting in motor defects.^61^ Although efforts at optimization generated ∼1500 potential variants, some with increased therapeutic safety windows, selectivity to reduce motor and muscle weakness deficits remained a challenge.^61^ With the increasing availability of peptide—VGIC structures, rational design of peptide toxin mutants is now possible (Fig. 6). Our own group previously utilized physics-based Rosetta methods^236^ and the structure of the ProTx2—hNa_V_1.7- Na_V_Ab complex, a human-bacterial chimera,^160^ to generate ProTx2 peptide analogs targeting hNa_V_1.7 with nanomolar potency and ∼1000-fold selectivity to hNa_V_1.1, hNa_V_1.3-1.5, hNa_V_1.8, and hNa_V_1.9.^62^ This design process was performed through iterative in silico peptide mutations, energy minimization, and ranking of the peptide—channel interface. We tested only 27 ProTx2 analogs over 4 design cycles derived from over 1 million in silico designed peptides, highlighting the potential of physics-based redesign for VGIC-selective targeting.

Beyond in silico protein interface redesign, binding free energy predictions based on structural models of peptide toxin–VGIC complexes represent another promising approach to support mutagenesis studies aimed at optimizing peptide toxins. In this strategy, detailed energetic predictions, often derived from MD simulations combined with free energy perturbation calculations can help quantify the contribution of individual residues to the binding interface as well as estimate the effect of mutations on the binding energies, allowing for prioritization of point mutations for experimental testing. These simulations can also be used to compare binding energies across homologous channels, enabling parallel evaluation of how specific mutations influence both target and off-target affinity, and thus guide the selection of variants with improved selectivity profiles. Although this approach has not yet been widely applied to large-scale toxin optimization campaigns, several studies have demonstrated its value in benchmarking toxin–channel interactions against available experimental data. These include analyses of ShK analogs binding to K_V_1.3,^237^ HsTX1 interactions with K_V_1 channels,^238^ and ProTx2 binding to Na_V_1.7.^239^ Such approaches offer a rational framework for navigating mutational space more efficiently and could ultimately reduce the number of experimental iterations required to achieve a desired pharmacological profile.

### E De novo protein design using deep learning

By integrating evolutionary sequence and structural data, it is now possible to accurately predict protein structures, including VGICs,^240^ using AlphaFold or other multimodal structure prediction models.^241^^,^^242^ With deep learning methods like RFdiffusion^243^ or BindCraft,^244^ atomic-precision design of de novo (ie, built from first principles, without templates or homologous sequences) protein binders tailored to specific targets has become feasible,^243^^,^^245^ bypassing the need for existing natural binder scaffolds, such as peptide toxins or antibodies, and enabling the creation of novel proteins from scratch, based exclusively on the target’s structure (Fig. 6). Although these methods have not yet been applied to ion channels, their potential for developing selective channel blockers and modulators is substantial as recently reviewed by us.^246^ Preliminary efforts toward the de novo design of protein-based ion channel modulators using these approaches have already been reported by academic labs at conferences.^247^, ^248^, ^249^ A recent de novo peptide modulator of Na_V_1.5 designed with these approaches was shown to reverse a GOF pathogenic phenotype associated with cardiac arrhythmia and epilepsy.^250^ This result provides a proof-of-concept for AI-enabled VGIC-targeting de novo protein binder design; yet, the intracellular binding site of this peptide modulator requires intracellular delivery, constraining its therapeutic translatability.

De novo protein binder design with deep learning can also benefit from available structural data of peptide toxins targeting VGICs. With methods like RFdiffusion,^243^^,^^251^^,^^252^ de novo peptides could be designed to adopt a similar binding mode as natural peptides, by extracting critical functional binding motifs of the natural peptide toxin while redesigning the surrounding backbone. Alternatively, fold-conditioning approaches can be used to design de novo binders with backbone conformations resembling inhibitor cystine knots, enabling the development of new functional motifs inspired by existing folds and expanding the accessible structure–function design space.

Beyond de novo binder design, deep learning models have also been adapted for antibody and nanobody design^253^ and for generating macrocycles.^254^ In the context of ion channels, therapeutic antibodies hold great promise due to their ability to engage specific extracellular domains with high affinity and precision.^10^^,^^105^ However, their development has traditionally been hampered by the complex conformational dynamics of ion channels and the transient exposure of key epitopes, as channels cycle through distinct functional states. With the ability to design against a specific state and target subtype-specific extracellular features, generative models have the potential to design antibodies that selectively target conformations of interest, such as loops exposed only during inactivation that differ across subtypes. This opens the door to the design of state-specific antibodies.

Macrocyclic peptides, meanwhile, occupy a unique physicochemical space between highly selective biologics and membrane permeable small molecules. They are synthetically accessible, allow for the incorporation of unnatural amino acids, and, like small molecules, are amenable to lead optimization via traditional medicinal chemistry efforts.^255^ For ion channels, their conformational rigidity could enable stable and specific engagement with challenging epitopes, such as VSDs or extracellular loops involved in gating, while their flexibility and small size permit passive diffusion across membranes, access to buried channel crevices, and oral availability. Recent examples of orally available, macrocyclic peptides that have reached clinical trials are the PCSK9 inhibiting peptide MK-0616^256^ and the interleukin-23 receptor-antagonist peptide JNJ-77242113.^257^ With generative models now able to design cyclic scaffolds de novo instead of identifying them through screening approaches,^254^ there is growing potential to create macrocycles that target VGIC with high selectivity and possibly even conformationally defined precision.

Together, these modalities, such as de novo binders, antibodies, nanobodies, and macrocycles, illustrate how deep learning can accelerate the development of protein-based therapeutics (Fig. 6). Realizing this potential will require close interaction between computational design, VS, and experimental validation.

## VII Conclusions and outlook

Ion channel drug discovery has often been described as difficult and challenging, and although none of the drug candidates targeting Na_V_, Ca_V,_ or K_V_ channels currently in clinical trials, had a particularly fast or easy development path, or is guaranteed to obtain approval, we believe that the many years of work devoted by researchers in academia and industry to studying ion channels are starting to pay-off and that there will be several approvals for drugs targeting VGICs before 2030. In addition to the drug candidates for VGICs we reviewed here, there are a substantial number of compounds targeting other ion channels in various stages of clinical trials (Table 4), also illustrating the emergence of ASOs and gene therapies as alternative treatment modalities to traditional small molecules. The transient receptor potential melastatin 8 (TRPM8) channel agonist acoltremon (TRYTYR) was approved in May 2025 for dry eye disease, where the compound increases tear production and induces a sensation of coolness.^258^

**Table 4:** Clinical Development candidates targeting other ion channels

Compound	Mechanism of Action	Indication	Company	Clinical Phase
Inaxaplin (VTX-147)	APOL1 inhibitor	APOL1-mediated kidney disease	Vertex Pharmaceuticals	Phase III
MZE829	APOL1 inhibitor	APOL1-mediated kidney disease	Maze Therapeutics	Phase II
KIO-301	Photo-switchable HCN and K_V_ blocker	Retinitis pigmentosa (Orphan Drug designation)	Kiora Pharmaceuticals	Phase II
KER-0193	K_Ca_1.1 (BK) modulator	Fragile X syndrome	Kaerus Biosciences	Phase II
AP30663	K_Ca_2 (SK) channel inhibitor	Acute cardioversion of atrial fibrillation	Acesion Pharma	Phase II
AP31969	K_Ca_2 (SK) channel inhibitor	Sinus rhythm maintenance	Acesion Pharma	Phase I
DES-7987	K_Ca_3.1 (IK) channel inhibitor	Asthma or stroke	DE Shaw Research	Phase I
CVN293	TWIK (K_2P_13.1) inhibitor	Frontotemporal Dementia	Cerevance	Phase I
DES-9384	TRPA1 inhibitor	Cough or rheumatoid arthritis	DE Shaw Research	Phase I
BHV-2100	TRPM3 antagonist	Pain disorder	Biohaven	Phase II
Alcotremon (AR-15512)	TRPM8 agonist	Drye eye disease	Alcon	FDA approval as TRYPTYR on May 29, 2025
IVW-1001	TRPM8 agonist	Drye eye disease	IView Therapeutics	Phase II
AX-8	TRPM8 agonist	Chronic cough	Axalbion	Phase IIa
Vocacapsaicin	TRPV1 agonist	Postsurgical analgesia	Concentric Analgesics	Phase II
Resiniferatoxin	TRPV1 agonist	Osteoarthritis knee pain	Grünenthal	Phase III
ACD440	TRPV1 antagonist	Erythromelalgia (Orphan Drug designation)	AlzeCure	Phase IIb/III
ABS-0871	TRPV4 inhibitor	Charcot Marie Tooth disease type 2C	Actio Biosciences	Phase I
Auxora	CRAC channel blocker	Acute pancreatitis	CalciMedica	Phase II completed
Auxora	CRAC channel blocker	Acute kidney injury	CalciMedica	Phase II
Camlipixant (NEO-5937)	P_2_X_3_ antagonist	Chronic cough	GSK	Phase III
ETD001	ENaC inhibitor	Cystic fibrosis	Enterprise Therapeutics	Phase IIa
Vanza triple (Vanzocaftor, Tezocaftor, Deutivacaftor)	CFTR chloride channel correctors and potentiators	Cystic fibrosis patients with at least 1 F508del mutation	Vertex Pharmaceuticals	FDA approval as ALYFTREK in Dec 2024
VX-522	CFTR chloride channel mRNA in lipid nanoparticles	Cystic fibrosis patients who make no CFTR protein	Vertex Pharmaceuticals	Phase I/II
BI 3720931	CFTR gene therapy	Cystic fibrosis; inhalable lentivirus	Boehringer Ingelheim	Phase I
NMD670	CLC1 inhibitor	Charcot Marie Tooth disease type 1 and 2	NMD Pharma	Phase IIa
NMD670	CLC1 inhibitor	Myasthenia gravis	NMD Pharma	Phase IIb
Cytisinicline	nAChR partial antagonist	Nicotine vaping cessation	Achieve Life Sciences	Phase III
BNC210	*α*7 nAChR PAM	Social anxiety in posttraumatic stress disorder	Neuphoria Therapeutics	Phase III
MK-1167	*α*7 nAChR NAM	Alzheimer’s disease	Neuphoria Therapeutics	
ENX-102	*α*2,3,5-GABA_AR_ specific PAM	Generalized anxiety disorder	Engrail Therapeutics	Phase II
ACP-711 (SAN711)	*α*3-GABA_AR_ specific PAM	ET	Acadia Pharmaceuticals	Phase IIb
SAN2219	*α*2,3,5-GABA_AR_ specific PAM	Epilepsy	Saniona	Phase I
AP-325	GABA_AR_ PAM	Postsurgical neuropathic pain	Algiax Therapeutics	Phase II
Fasedienol	GABA_AR_ PAM	Social anxiety disorder (Fast track designation)	Vistagen	Phase III
SAN2465	*α*-GABA_AR_ specific NAM	Major depressive disorder	Saniona	Phase I
Danegaptide	Connexin 43 opener	Diabetic retinopathy	Breye Therapeutics	Phase Ib
Nexagon	Connexin 43 ASO	Persistent corneal epithelial defects following severe ocular injuries	Amber Ophthalmics	Phase II
Apimostinel (GATE-202)	NMDA_R_ PAM	MDD	Syndeio Biosciences	Phase II
GM-1020	NMDA_R_ antagonist	MDD	Gilgamesh Pharma	Phase IIa
NBI-1070770	GluR NMDA NR2B-selective NAM	MDD	Neurocrine Bioscience	Phase II
NBI-10605845	AMPA GluR PAM	MDD	Neurocrine Bioscience	Phase II
RAP-219	AMPA GluR Tarp *γ*8 subunit antagonist	Focal epilepsy	Rapport Therapeutics	Phase II
ES 481	AMPA GluR Tarp *γ*8 subunit antagonist	Drug resistant epilepsy	ES Therapeutics	Phase II

ASO, antisense oligonucleotide; ET, essential tremor; PAM, positive allosteric modulator; MDD, major depressive disorder; NAM, negative allosteric modulator.

The above-described computational methods are likely to increasingly integrate with experimental screening workflows, guiding library design, reducing attrition rates, and ultimately lowering the cost of identifying hits and accelerating the early stages of VGIC drug discovery. We predict that deep learning accelerated VS of small molecules will likely replace target-based ultra-high-throughput experimental screening and make a perfect combination with medium-throughput automated electrophysiology in VGIC drug discovery programs. At this point, no AI-enabled drug candidate modulating VGIC has made it into the clinic. However, the Traf2- and Nck-interacting kinase inhibitor rentosertib recently finished a phase IIa clinical trial for idiopathic pulmonary fibrosis in which it demonstrated an acceptable safety profile and early signs of efficacy.^259^ Both the target and the compound were identified using Insilico Medicine’s generative AI-based biology and chemistry platforms demonstrating the potential impact of using AI in drug discovery.^260^

For protein-based therapeutics, we believe that deep learning methods have the potential to significantly aid in the development of antibodies and nanobodies, which have been challenging for VGICs, and enable the design of de novo proteins and macrocycles—novel modalities that so far have not been utilized in ion channel drug development.

However, it should be noted that even the most advanced and exciting new technologies cannot replace the fundamental determinants of success in drug development, which ultimately depends on well-validated targets, careful optimization of selectivity and pharmacokinetic properties, and the sustained, rigorous effort required to translate a lead compound into a viable therapeutic. As the examples of K_V_7.2/K_V_7.3, K_V_1.3, and Ca_V_3 channels in our review illustrate, revisiting “old” targets with new molecules and well-chosen clinical indications could be a successful strategy.

## Conflicts of interest

No author has an actual or perceived conflict of interest with the contents of this article.

## References

[1] Alexander S.P.H., Mathie A.A., Peters J.A. (2023). The concise guide to PHARMACOLOGY 2023/24: ion channels. Br J Pharmacol.

[2] Osteen J.D., Immani S., Tapley T.L. (2025). Pharmacology and mechanism of action of suzetrigine, a potent and selective Na_V_1.8 pain signal inhibitor for the treatment of moderate to severe pain. Pain Ther.

[3] Tringham E., Powell K.L., Cain S.M. (2012). T-type calcium channel blockers that attenuate thalamic burst firing and suppress absence seizures. Sci Transl Med.

[4] Pennington M.W., Beeton C., Galea C.A. (2009). Engineering a stable and selective peptide blocker of the Kv1.3 channel in T lymphocytes. Mol Pharmacol.

[5] Carotenuto L., Keminer O., Carleo G. (Published online July 23, 2025). The fast-dissociating D_2_ antagonist antipsychotic JNJ-37822681 is a neuronal Kv7 channel opener: Potential repurposing for epilepsy treatment. Br J Pharmacol.

[6] Oyrer J., Maljevic S., Scheffer I.E., Berkovic S.F., Petrou S., Reid C.A. (2018). Ion channels in genetic epilepsy: from genes and mechanisms to disease-targeted therapies. Pharmacol Rev.

[7] Catterall W.A., Lenaeus M.J., Gamal El-Din T.M. (2020). Structure and pharmacology of voltage-gated sodium and calcium channels. Annu Rev Pharmacol Toxicol.

[8] Mantegazza M., Cestele S., Catterall W.A. (2021). Sodium channelopathies of skeletal muscle and brain. Physiol Rev.

[9] Huang J., Pan X., Yan N. (2024). Structural biology and molecular pharmacology of voltage-gated ion channels. Nat Rev Mol Cell Biol.

[10] Wulff H., Christophersen P., Colussi P., Chandy K.G., Yarov-Yarovoy V. (2019). Antibodies and venom peptides: new modalities for ion channels. Nat Rev Drug Discov.

[11] Wulff H., Castle N.A., Pardo L.A. (2009). Voltage-gated potassium channels as therapeutic targets. Nat Rev Drug Discov.

[12] Harrison C. (2025). Vertex's opioid-free drug for acute pain wins FDA approval. Nat Biotechnol.

[13] Fertig N., Farre C. (2010). Renaissance of ion channel research and drug discovery by patch clamp automation. Future Med Chem.

[14] Kaczorowski G.J., McManus O.B., Priest B.T., Garcia M.L. (2008). Ion channels as drug targets: the next GPCRs. J Gen Physiol.

[15] Santos R., Ursu O., Gaulton A. (2017). A comprehensive map of molecular drug targets. Nat Rev Drug Discov.

[16] Alexander S.P., Striessnig J., Kelly E. (2017). THE CONCISE GUIDE TO PHARMACOLOGY 2017/18: voltage-gated ion channels. Br J Pharmacol.

[17] Alexander S.P., Peters J.A., Kelly E. (2017). THE CONCISE GUIDE TO PHARMACOLOGY 2017/18: ligand-gated ion channels. Br J Pharmacol.

[18] Alexander S.P., Kelly E., Marrion N.V. (2017). THE CONCISE GUIDE TO PHARMACOLOGY 2017/18: other ion channels. Br J Pharmacol.

[19] Hille B. (2001).

[20] Catterall W.A. (2017). Forty years of sodium channels: structure, function, pharmacology, and epilepsy. Neurochem Res.

[21] Grandi E., Sanguinetti M.C., Bartos D.C. (2017). Potassium channels in the heart: structure, function and regulation. J Physiol.

[22] Stephens G., Stevens E. (2024).

[23] Catterall W.A. (2023). Voltage gated sodium and calcium channels: discovery, structure, function, and Pharmacology. Channels.

[24] Alsaloum M., Dib-Hajj S.D., Page D.A., Ruben P.C., Krainer A.R., Waxman S.G. (2025). Voltage-gated sodium channels in excitable cells as drug targets. Nat Rev Drug Discov.

[25] Cox J.J., Reimann F., Nicholas A.K. (2006). An SCN9A channelopathy causes congenital inability to experience pain. Nature.

[26] McCormack K., Santos S., Chapman M.L. (2013). Voltage sensor interaction site for selective small molecule inhibitors of voltage-gated sodium channels. Proc Natl Acad Sci USA.

[27] Ahuja S., Mukund S., Deng L. (2015). Structural basis of Nav1.7 inhibition by an isoform-selective small-molecule antagonist. Science.

[28] Bennett D.L., Clark A.J., Huang J., Waxman S.G., Dib-Hajj S.D. (2019). The role of voltage-gated sodium channels in pain signaling. Physiol Rev.

[29] Payandeh J., Hackos D.H. (2018). Selective ligands and drug discovery targeting the voltage-gated sodium channel Nav1.7. Handb Exp Pharmacol.

[30] Mulcahy J.V., Pajouhesh H., Beckley J.T., Delwig A., Du Bois J., Hunter J.C. (2019). Challenges and opportunities for therapeutics targeting the voltage-gated sodium channel isoform Na_V_1.7. J Med Chem.

[31] Xie Y.F., Yang J., Ratte S., Prescott S.A. (2024). Similar excitability through different sodium channels and implications for the analgesic efficacy of selective drugs. eLife.

[32] Wengert E.R., Wagley P.K., Strohm S.M. (2022). Targeted augmentation of nuclear gene output (TANGO) of *Scn1a* rescues parvalbumin interneuron excitability and reduces seizures in a mouse model of Dravet syndrome. Brain Res.

[33] Bialer M., Johannessen S.I., Koepp M.J. (2024). Progress report on new medications for seizures and epilepsy: a summary of the 17th Eilat Conference on New Antiepileptic Drugs and Devices (EILAT XVII). II. Drugs in more advanced clinical development. Epilepsia.

[34] Carvill G.L., Engel K.L., Ramamurthy A. (2018). Aberrant inclusion of a poison exon causes Dravet syndrome and related SCN1A-associated genetic epilepsies. Am J Hum Genet.

[35] Isom L.L., Knupp K.G. (2021). Dravet syndrome: novel approaches for the most common genetic epilepsy. Neurotherapeutics.

[36] Perucca E., White H.S., Bialer M. (2023). New GABA-targeting therapies for the treatment of seizures and epilepsy: II. Treatments in clinical development. CNS Drugs.

[37] Kahlig K.M., Scott L., Hatch R.J. (2022). The novel persistent sodium current inhibitor PRAX-562 has potent anticonvulsant activity with improved protective index relative to standard of care sodium channel blockers. Epilepsia.

[38] Wagner M., Berecki G., Fazeli W. (2025). Antisense oligonucleotide treatment in a preterm infant with early-onset SCN2A developmental and epileptic encephalopathy. Nat Med.

[39] Dib-Hajj S.D., Waxman S.G. (2019). Sodium channels in human pain disorders: genetics and pharmacogenomics. Annu Rev Neurosci.

[40] Eagles D.A., Chow C.Y., King G.F. (2022). Fifteen years of Na_V_1.7 channels as an analgesic target: why has excellent in vitro pharmacology not translated into in vivo analgesic efficacy?. Br J Pharmacol.

[41] McDonnell A., Collins S., Ali Z. (2018). Efficacy of the Nav1.7 blocker PF-05089771 in a randomised, placebo-controlled, double-blind clinical study in subjects with painful diabetic peripheral neuropathy. Pain.

[42] Bankar G., Goodchild S.J., Howard S. (2018). Selective Na_V_1.7 antagonists with long residence time show improved efficacy against inflammatory and neuropathic pain. Cell Rep.

[43] Safina B.S., McKerrall S.J., Sun S. (2021). Discovery of acyl-sulfonamide Nav1.7 inhibitors GDC-0276 and GDC-0310. J Med Chem.

[44] Rothenberg M.E., Tagen M., Chang J.H. (2019). Safety, tolerability, and pharmacokinetics of GDC-0276, a novel Na_V_1.7 inhibitor, in a first-in-human, single- and multiple-dose study in healthy volunteers. Clin Drug Investig.

[45] Mulcahy J.V., Beckley J.T., Klas S.D. (2024). ST-2560, a selective inhibitor of the Na_V_1.7 sodium channel, affects nocifensive and cardiovascular reflexes in non-human primates. Br J Pharmacol.

[46] Deng L., Dourado M., Reese R.M. (2023). Nav1.7 is essential for nociceptor action potentials in the mouse in a manner independent of endogenous opioids. Neuron.

[47] Moreno A.M., Aleman F., Catroli G.F. (2021). Long-lasting analgesia via targeted in situ repression of Na_V_1.7 in mice. Sci Transl Med.

[48] Brazil R. (2023). Peptide nucleic acids promise new therapeutics and gene editing tools. ACS Cent Sci.

[49] Gomez K., Stratton H.J., Duran P. (2023). Identification and targeting of a unique Na_V_1.7 domain driving chronic pain. Proc Natl Acad Sci USA.

[50] Jones J., Correll D.J., Lechner S.M. (2023). Selective inhibition of Na_V_1.8 with VX-548 for acute pain. N Engl J Med.

[51] Hu S., Lyu D., Gao J. (2025). Suzetrigine: the first Nav1.8 inhibitor approved for the treatment of moderate to severe acute pain. Drug Discov Ther.

[52] Gilchrist J.M., Yang N.D., Jiang V., Moyer B.D. (2024). Pharmacologic characterization of LTGO-33, a selective small molecule inhibitor of the voltage-gated sodium channel Na_V_1.8 with a unique mechanism of action. Mol Pharmacol.

[53] Omana-Zapata I., Khabbaz M.A., Hunter J.C., Clarke D.E., Bley K.R. (1997). Tetrodotoxin inhibits neuropathic ectopic activity in neuromas, dorsal root ganglia and dorsal horn neurons. Pain.

[54] Gonzalez-Cano R., Ruiz-Cantero M.C., Santos-Caballero M., Gomez-Navas C., Tejada M.A., Nieto F.R. (2021). Tetrodotoxin, a potential drug for neuropathic and cancer pain relief?. Toxins.

[55] Lyu Y.S., Park S.K., Chung K., Chung J.M. (2000). Low dose of tetrodotoxin reduces neuropathic pain behaviors in an animal model. Brain Res.

[56] Hagen N.A., Cantin L., Constant J. (2017). Tetrodotoxin for moderate to severe cancer-related pain: a multicentre, randomized, double-blind, placebo-controlled, parallel-design trial. Pain Res Manag.

[57] Genevois A.L., Ruel J., Penalba V. (2021). Analgesic effects of topical amitriptyline in patients with chemotherapy-induced peripheral neuropathy: mechanistic insights from studies in mice. J Pain.

[58] Singh R., Hahn M.K., Bansal Y., Agarwal S.M., Remington G. (2024). Evenamide: a potential pharmacotherapeutic alternative for treatment-resistant schizophrenia. Int J Neuropsychopharmacol.

[59] Zhang Y., Ding Y., Zeng Z. (2024). Intra-channel bi-epitopic crosslinking unleashes ultrapotent antibodies targeting Na_V_1.7 for pain alleviation. Cell Rep Med.

[60] Cohen F., Yuan H., DePoy E.M.G., Silberstein S.D. (2022). The arrival of anti-CGRP monoclonal antibodies in migraine. Neurotherapeutics.

[61] Flinspach M., Xu Q., Piekarz A.D. (2017). Insensitivity to pain induced by a potent selective closed-state Nav1.7 inhibitor. Sci Rep.

[62] Nguyen P.T., Nguyen H.M., Wagner K.M. (2022). Computational design of peptides to target Na_V_1.7 channel with high potency and selectivity for the treatment of pain. Elife.

[63] Qin S., Tang X., Chen Y. (2022). mRNA-based therapeutics: powerful and versatile tools to combat diseases. Signal Transduct Target Ther.

[64] Wang L., Wang N., Zhang W. (2022). Therapeutic peptides: current applications and future directions. Signal Transduct Target Ther.

[65] Stambler B.S., Dorian P., Sager P.T. (2018). Etripamil nasal spray for rapid conversion of supraventricular tachycardia to sinus rhythm. J Am Coll Cardiol.

[66] Stambler B.S., Camm A.J., Alings M. (2023). Self-administered intranasal etripamil using a symptom-prompted, repeat-dose regimen for atrioventricular-nodal-dependent supraventricular tachycardia (RAPID): a multicentre, randomised trial. Lancet.

[67] Stambler B.S., Plat F., Sager P.T. (2022). First randomized, multicenter, placebo-controlled study of self-administered intranasal etripamil for acute conversion of spontaneous paroxysmal supraventricular tachycardia (NODE-301). Circ Arrhythm Electrophysiol.

[68] Jha M., Song D., Kung A. (2025). Efficacy and safety of intranasal etripamil for paroxysmal supraventricular tachycardia: meta-analysis of randomized controlled trials. J Clin Med.

[69] Ip J.E., Coutu B., Bennett M.T. (2023). Etripamil nasal spray for conversion of repeated spontaneous episodes of paroxysmal supraventricular tachycardia during long-term follow-up: results from the NODE-302 study. J Am Heart Assoc.

[70] Po A.L., Zhang W.Y. (1998). What lessons can be learnt from withdrawal of mibefradil from the market?. Lancet.

[71] Zhao Y., Huang G., Wu Q. (2019). Cryo-EM structures of apo and antagonist-bound human Cav3.1. Nature.

[72] LeBlanc B.W., Lii T.R., Huang J.J. (2016). T-type calcium channel blocker Z944 restores cortical synchrony and thalamocortical connectivity in a rat model of neuropathic pain. Pain.

[73] Harding E.K., Dedek A., Bonin R.P., Salter M.W., Snutch T.P., Hildebrand M.E. (2021). The T-type calcium channel antagonist, Z944, reduces spinal excitability and pain hypersensitivity. Br J Pharmacol.

[74] Scott L., Puryear C.B., Belfort G.M. (2022). Translational pharmacology of PRAX-944, a novel T-type calcium channel blocker in development for the treatment of essential tremor. Mov Disord.

[75] Gomez K., Santiago U., Nelson T.S. (2023). A peptidomimetic modulator of the Ca_V_2.2 N-type calcium channel for chronic pain. Proc Natl Acad Sci USA.

[76] Allen H.N., Hestehave S., Duran P., Nelson T.S., Khanna R. (2024). Uncoupling the CRMP2-Ca_V_2.2 interaction reduces pain-like behavior in a preclinical joint-pain model. J Pain.

[77] Garcia-Caballero A., Gadotti V.M., Chen L., Zamponi G.W. (2016). A cell-permeant peptide corresponding to the cUBP domain of USP5 reverses inflammatory and neuropathic pain. Mol Pain.

[78] Garcia-Caballero A., Gadotti V.M., Ali M.Y. (2022). A synthetically accessible small-molecule inhibitor of USP5-Cav3.2 calcium channel interactions with analgesic properties. ACS Chem Neurosci.

[79] Patel K.V., Gadotti V.M., Garcia-Caballero A. (2024). Development of tetrahydroquinoline-based inhibitors for chronic pain. ACS Chem Neurosci.

[80] Johnston J., Forsythe I.D., Kopp-Scheinpflug C. (2010). Going native: voltage-gated potassium channels controlling neuronal excitability. J Physiol.

[81] Sanguinetti M.C., Tristani-Firouzi M. (2006). hERG potassium channels and cardiac arrhythmia. Nature.

[82] Sato T., Yuki H., Ogura K., Honma T. (2018). Construction of an integrated database for hERG blocking small molecules. PLoS One.

[83] DeCoursey T.E., Chandy K.G., Gupta S., Cahalan M.D. (1984). Voltage-gated K^+^ channels in human T lymphocytes: a role in mitogenesis?. Nature.

[84] Matteson D.R., Deutsch C. (1984). K channels in T lymphocytes: a patch clamp study using monoclonal antibody adhesion. Nature.

[85] Cahalan M.D., Chandy K.G. (2009). The functional network of ion channels in T lymphocytes. Immunol Rev.

[86] Feske S., Wulff H., Skolnik E.Y. (2015). Ion channels in innate and adaptive immunity. Annu Rev Immunol.

[87] Chandy K.G., Wulff H., Beeton C., Pennington M., Gutman G.A., Cahalan M.D. (2004). K+ channels as targets for specific immunomodulation. Trends Pharmacol Sci.

[88] Koo G.C., Blake J.T., Shah K. (1999). Correolide and derivatives are novel immunosuppressants blocking the lymphocyte Kv1.3 potassium channels. Cell Immunol.

[89] Miao S., Bao J., Garcia M.L. (2003). Benzamide derivatives as blockers of the Kv1.3 ion channel. Bioorg Med Chem Lett.

[90] Wulff H., Calabresi P.A., Allie R. (2003). The voltage-gated Kv1.3 K^+^ channel in effector memory T cells as new target for MS. J Clin Invest.

[91] Beeton C., Wulff H., Standifer N.E. (2006). Kv1.3 channels are a therapeutic target for T cell-mediated autoimmune diseases. Proc Natl Acad Sci USA.

[92] Matheu M.P., Beeton C., Garcia A. (2008). Imaging of effector memory T cells during a delayed-type hypersensitivity reaction and suppression by Kv1.3 channel block. Immunity.

[93] Wulff H., Pennington M. (2007). Targeting effector memory T-cells with Kv1.3 blockers. Curr Opin Drug Discov Devel.

[94] Beeton C., Pennington M.W., Wulff H. (2005). Targeting effector memory T cells with a selective peptide inhibitor of Kv1.3 channels for therapy of autoimmune diseases. Mol Pharmacol.

[95] Tarcha E.J., Chi V., Munoz-Elias E.J. (2012). Durable pharmacological responses from the peptide drug ShK-186, a specific Kv1.3 channel inhibitor that suppresses T cell mediators of autoimmune disease. J Pharmacol Exp Ther.

[96] Chi V., Pennington M.W., Norton R.S. (2012). Development of a sea anemone toxin as an immunomodulator for therapy of autoimmune diseases. Toxicon.

[97] Tarcha E.J., Olsen C.M., Probst P. (2017). Safety and pharmacodynamics of dalazatide, a Kv1.3 channel inhibitor, in the treatment of plaque psoriasis: a randomized phase 1b trial. PLoS One.

[98] Mozaffar T., Wencel M., Goyal N., Philips C., Olsen C. (2017). Kv1.3 expression on effector memory T cells in sporadic inclusion body myositis: potential for targeted immunotherapy with dalazatide. Neuromuscul Disord.

[99] Tanner M.R., Tajhya R.B., Huq R. (2017). Prolonged immunomodulation in inflammatory arthritis using the selective Kv1.3 channel blocker HsTX1[R14A] and its PEGylated analog. Clin Immunol.

[100] Zhang H.K., Du M.J., Xie J. (2016). Autocrine-based selection of drugs that target ion channels from combinatorial venom peptide libraries. Angew Chem Int Ed Engl.

[101] Edwards W., Fung-Leung W.P., Huang C. (2014). Targeting the ion channel Kv1.3 with scorpion venom peptides engineered for potency, selectivity, and half-life. J Biol Chem.

[102] Murray J.K., Qian Y.X., Liu B. (2015). Pharmaceutical optimization of peptide toxins for ion channel targets: potent, selective, and long-lived antagonists of Kv1.3. J Med Chem.

[103] Fung-Leung W.P., Edwards W., Liu Y. (2017). T cell subset and stimulation strength-dependent modulation of T cell activation by Kv1.3 blockers. PLoS One.

[104] Chiang E.Y., Li T., Jeet S. (2017). Potassium channels Kv1.3 and KCa3.1 cooperatively and compensatorily regulate antigen-specific memory T cell functions. Nat Commun.

[105] Bednenko J., Harriman R., Marien L. (2018). A multiplatform strategy for the discovery of conventional monoclonal antibodies that inhibit the voltage-gated potassium channel Kv1.3. MAbs.

[106] Stortelers C., Pinto-Espinoza C., Van Hoorick D., Koch-Nolte F. (2018). Modulating ion channel function with antibodies and nanobodies. Curr Opin Immunol.

[107] Wang R.S.E., Wang Y., Zhang Y.H. (2016). Rational design of a Kv1.3 channel-blocking antibody as a selective immunosuppressant. Proc Natl Acad Sci USA.

[108] Bell D.C., Karratt-Vellatt A., Surade S. (2018). Knotbodies: a new generation of ion channel therapeutic biologics created by fusing knottin toxins into antibodies. Biophys J.

[109] Nguyen W., Howard B.L., Neale D.S. (2010). Use of Kv1.3 blockers for inflammatory skin conditions. Curr Med Chem.

[110] Gubic S., Hendrickx L.A., Toplak Z. (2021). Discovery of K_V_1.3 ion channel inhibitors: medicinal chemistry approaches and challenges. Med Res Rev.

[111] Schmitz A., Sankaranarayanan A., Azam P. (2005). Design of PAP-1, a selective small molecule Kv1.3 blocker, for the suppression of effector memory T cells in autoimmune diseases. Mol Pharmacol.

[112] Jensen M.O., Borhani D.W., Lindorff-Larsen K. (2010). Principles of conduction and hydrophobic gating in K^+^ channels. Proc Natl Acad Sci USA.

[113] Jensen M.O., Jogini V., Borhani D.W., Leffler A.E., Dror R.O., Shaw D.E. (2012). Mechanism of voltage gating in potassium channels. Science.

[114] Unterweger A.L., Jensen M.O., Giordanetto F. (2021). Suppressing Kv1.3 ion channel activity with a novel small molecule inhibitor ameliorates inflammation in a humanised mouse model of ulcerative colitis. J Crohns Colitis.

[115] Kaczmarek L.K., Zhang Y. (2017). Kv3 channels: enablers of rapid firing, neurotransmitter release, and neuronal endurance. Physiol Rev.

[116] Muona M., Berkovic S.F., Dibbens L.M. (2015). A recurrent de novo mutation in KCNC1 causes progressive myoclonus epilepsy. Nat Genet.

[117] Nascimento F.A., Andrade D.M. (2016). Myoclonus epilepsy and ataxia due to potassium channel mutation (MEAK) is caused by heterozygous KCNC1 mutations. Epileptic Disord.

[118] Zhang Y., Kaczmarek L.K. (2016). Kv3.3 potassium channels and spinocerebellar ataxia. J Physiol.

[119] Faulkner I.E., Pajak R.Z., Harte M.K., Glazier J.D., Hager R. (2024). Voltage-gated potassium channels as a potential therapeutic target for the treatment of neurological and psychiatric disorders. Front Cell Neurosci.

[120] Boddum K., Hougaard C., Xiao-Ying Lin J. (2017). Kv3.1/Kv3.2 channel positive modulators enable faster activating kinetics and increase firing frequency in fast-spiking GABAergic interneurons. Neuropharmacology.

[121] Rosato-Siri M.D., Zambello E., Mutinelli C. (2015). A novel modulator of Kv3 potassium channels regulates the firing of parvalbumin-positive cortical interneurons. J Pharmacol Exp Ther.

[122] Liang Q., Chi G., Cirqueira L. (2024). The binding and mechanism of a positive allosteric modulator of Kv3 channels. Nat Commun.

[123] Chen Y.T., Hong M.R., Zhang X.J. (2023). Identification, structural, and biophysical characterization of a positive modulator of human Kv3.1 channels. Proc Natl Acad Sci USA.

[124] Botte M., Huber S., Bucher D. (2022). Apo and ligand-bound high resolution Cryo-EM structures of the human Kv3.1 channel reveal a novel binding site for positive modulators. PNAS Nexus.

[125] Hall D.A., Ray J., Watson J. (2019). A balanced randomised placebo controlled blinded phase IIa multi-centre study to investigate the efficacy and safety of AUT00063 versus placebo in subjective tinnitus: the QUIET-1 trial. Hear Res.

[126] Angelescu I., Kaar S.J., Marques T.R. (2022). The effect of AUT00206, a Kv3 potassium channel modulator, on dopamine synthesis capacity and the reliability of [^18^F]-FDOPA imaging in schizophrenia. J Psychopharmacol.

[127] Musselman M., Huynh E., Kelshikar R., Lee E., Malik M., Faden J. (2023). Potassium channel modulators and schizophrenia: an overview of investigational drugs. Exp Opin Investig Drugs.

[128] Parekh P.K., Sidor M.M., Gillman A. (2018). Antimanic efficacy of a novel Kv3 potassium channel modulator. Neuropsychopharmacology.

[129] Delmas P., Brown D.A. (2005). Pathways modulating neural KCNQ/M (Kv7) potassium channels. Nat Rev Neurosci.

[130] Jespersen T., Grunnet M., Olesen S.P. (2005). The KCNQ1 potassium channel: from gene to physiological function. Physiology.

[131] Sanguinetti M.C., Curran M.E., Zou A. (1996). Coassembly of K(V)LQT1 and minK (IsK) proteins to form cardiac I(Ks) potassium channel. Nature.

[132] Lynch J.J., Houle M.S., Stump G.L. (1999). Antiarrhythmic efficacy of selective blockade of the cardiac slowly activating delayed rectifier current, I(Ks), in canine models of malignant ischemic ventricular arrhythmia. Circulation.

[133] So P.P., Hu X.D., Backx P.H., Puglisi J.L., Dorian P. (2006). Blockade of IKs by HMR 1556 increases the reverse rate-dependence of refractoriness prolongation by dofetilide in isolated rabbit ventricles. Br J Pharmacol.

[134] Bauer A., Koch M., Kraft P. (2005). The new selective I(Ks)-blocking agent HMR 1556 restores sinus rhythm and prevents heart failure in pigs with persistent atrial fibrillation. Basic Res Cardiol.

[135] Brown D.A., Passmore G.M. (2009). Neural KCNQ (Kv7) channels. Br J Pharmacol.

[136] Biervert C., Schroeder B.C., Kubisch C. (1998). A potassium channel mutation in neonatal human epilepsy. Science.

[137] Singh N.A., Charlier C., Stauffer D. (1998). A novel potassium channel gene, KCNQ2, is mutated in an inherited epilepsy of newborns. Nat Genet.

[138] Neubauer B.A., Waldegger S., Heinzinger J. (2008). KCNQ2 and KCNQ3 mutations contribute to different idiopathic epilepsy syndromes. Neurology.

[139] Goto A., Ishii A., Shibata M., Ihara Y., Cooper E.C., Hirose S. (2019). Characteristics of KCNQ2 variants causing either benign neonatal epilepsy or developmental and epileptic encephalopathy. Epilepsia.

[140] Borgini M., Mondal P., Liu R., Wipf P. (2021). Chemical modulation of Kv7 potassium channels. RSC Med Chem.

[141] Redford K.E., Abbott G.W. (2022). KCNQ potassium channels as targets of botanical folk medicines. Annu Rev Pharmacol Toxicol.

[142] Perucca E., Taglialatela M. (2025). Targeting Kv7 potassium channels for epilepsy. CNS Drugs.

[143] Wainger B.J., Macklin E.A., Vucic S. (2021). Effect of ezogabine on cortical and spinal motor neuron excitability in amyotrophic lateral sclerosis: a randomized clinical trial. JAMA Neurol.

[144] French J.A., Porter R.J., Perucca E. (2023). Efficacy and safety of XEN1101, a novel potassium channel opener, in adults with focal epilepsy: a phase 2b randomized clinical trial. JAMA Neurol.

[145] Xia X., Zhang Q., Jia Y. (2020). Molecular basis and restoration of function deficiencies of Kv7.4 variants associated with inherited hearing loss. Hear Res.

[146] Leitner M.G., Feuer A., Ebers O., Schreiber D.N., Halaszovich C.R., Oliver D. (2012). Restoration of ion channel function in deafness-causing KCNQ4 mutants by synthetic channel openers. Br J Pharmacol.

[147] Jiang Y., Lee A., Chen J. (2003). X-ray structure of a voltage-dependent K^+^ channel. Nature.

[148] Long S.B., Campbell E.B., Mackinnon R. (2005). Crystal structure of a mammalian voltage-dependent Shaker family K^+^ channel. Science.

[149] Long S.B., Campbell E.B., Mackinnon R. (2005). Voltage sensor of Kv1.2: structural basis of electromechanical coupling. Science.

[150] Li Z., Wu Q., Yan N. (2024). A structural atlas of druggable sites on Na_v_ channels. Channels.

[151] Payandeh J., Scheuer T., Zheng N., Catterall W.A. (2011). The crystal structure of a voltage-gated sodium channel. Nature.

[152] Shen H., Zhou Q., Pan X., Li Z., Wu J., Yan N. (2017). Structure of a eukaryotic voltage-gated sodium channel at near-atomic resolution. Science.

[153] Yan Z., Zhou Q., Wang L. (2017). Structure of the Nav1.4-β1 complex from electric eel. Cell.

[154] Pan X., Li Z., Zhou Q. (2018). Structure of the human voltage-gated sodium channel Na_v_ 1.4 in complex with β1. Science.

[155] West J.W., Patton D.E., Scheuer T., Wang Y., Goldin A.L., Catterall W.A. (1992). A cluster of hydrophobic amino acid residues required for fast Na^+^-channel inactivation. Proc Natl Acad Sci USA.

[156] Capes D.L., Goldschen-Ohm M.P., Arcisio-Miranda M., Bezanilla F., Chanda B. (2013). Domain IV voltage-sensor movement is both sufficient and rate limiting for fast inactivation in sodium channels. J Gen Physiol.

[157] Catterall W.A. (2012). Voltage-gated sodium channels at 60: structure, function and pathophysiology. J Physiol.

[158] Clairfeuille T., Cloake A., Infield D.T. (2019). Structural basis of α-scorpion toxin action on Na_v_ channels. Science.

[159] Shen H., Liu D., Wu K., Lei J., Yan N. (2019). Structures of human Nav1.7 channel in complex with auxiliary subunits and animal toxins. Science.

[160] Xu H., Li T., Rohou A. (2019). Structural basis of Nav1.7 inhibition by a gating-modifier spider toxin. Cell.

[161] Jiang D., Shi H., Tonggu L. (2020). Structure of the cardiac sodium channel. Cell.

[162] Huang X., Jin X., Huang G. (2022). Structural basis for high-voltage activation and subtype-specific inhibition of human Nav1.8. Proc Natl Acad Sci USA.

[163] Li Z., Jin X., Wu T. (2021). Structure of human Nav1.5 reveals the fast inactivation-related segments as a mutational hotspot for the long QT syndrome. Proc Natl Acad Sci USA.

[164] Chen H., Xia Z., Dong J. (2024). Structural mechanism of voltage-gated sodium channel slow inactivation. Nat Commun.

[165] Wu J., Yan Z., Li Z. (2015). Structure of the voltage-gated calcium channel Cav1.1 complex. Science.

[166] Zhao Y., Huang G., Wu J. (2019). Molecular basis for ligand modulation of a mammalian voltage-gated Ca^2+^ channel. Cell.

[167] Gao S., Yao X., Yan N. (2021). Structure of human Cav2.2 channel blocked by the painkiller ziconotide. Nature.

[168] Yao X., Gao S., Yan N. (2022). Structural basis for pore blockade of human voltage-gated calcium channel Cav1.3 by motion sickness drug cinnarizine. Cell Res.

[169] Yao X., Wang Y., Wang Z. (2022). Structures of the R-type human Cav2.3 channel reveal conformational crosstalk of the intracellular segments. Nat Commun.

[170] He L., Yu Z., Geng Z. (2022). Structure, gating, and pharmacology of human Ca_V_3.3 channel. Nat Commun.

[171] Gao S., Yao X., Chen J. (2023). Structural basis for human Cav1.2 inhibition by multiple drugs and the neurotoxin calciseptine. Cell.

[172] Wei Y., Yu Z., Wang L. (2024). Structural bases of inhibitory mechanism of Ca_V_1.2 channel inhibitors. Nat Commun.

[173] Huang J., Fan X., Jin X. (2024). Structural basis for human Cav3.2 inhibition by selective antagonists. Cell Res.

[174] Li Z., Cong Y., Wu T. (2024). Structural basis for different omega-agatoxin IVA sensitivities of the P–type and Q-type Cav2.1 channels. Cell Res.

[175] Chen Z., Mondal A., Abderemane-Ali F. (2023). EMC chaperone-Ca_V_ structure reveals an ion channel assembly intermediate. Nature.

[176] Wang W., MacKinnon R. (2017). Cryo-EM Structure of the open human ether-a-go-go-related K^+^ channel hERG. Cell.

[177] Sun J., MacKinnon R. (2020). Structural basis of human KCNQ1 modulation and gating. Cell.

[178] Li X., Zhang Q., Guo P. (2021). Molecular basis for ligand activation of the human KCNQ2 channel. Cell Res.

[179] Li T., Wu K., Yue Z., Wang Y., Zhang F., Shen H. (2021). Structural basis for the modulation of human KCNQ4 by small-molecule drugs. Mol Cell.

[180] Chi G., Liang Q., Sridhar A. (2022). Cryo-EM structure of the human Kv3.1 channel reveals gating control by the cytoplasmic T1 domain. Nat Commun.

[181] Reddi R., Matulef K., Riederer E.A., Whorton M.R., Valiyaveetil F.I. (2022). Structural basis for C-type inactivation in a Shaker family voltage-gated K^+^ channel. Sci Adv.

[182] Selvakumar P., Fernandez-Marino A.I., Khanra N. (2022). Structures of the T cell potassium channel Kv1.3 with immunoglobulin modulators. Nat Commun.

[183] Tyagi A., Ahmed T., Jian S. (2022). Rearrangement of a unique Kv1.3 selectivity filter conformation upon binding of a drug. Proc Natl Acad Sci U S A.

[184] Fernandez-Marino A.I., Tan X.F., Bae C., Huffer K., Jiang J., Swartz K.J. (2023). Inactivation of the Kv2.1 channel through electromechanical coupling. Nature.

[185] Mandala V.S., MacKinnon R. (2022). Voltage-sensor movements in the Eag Kv channel under an applied electric field. Proc Natl Acad Sci U S A.

[186] Mandala V.S., MacKinnon R. (2023). The membrane electric field regulates the PIP_2_-binding site to gate the KCNQ1 channel. Proc Natl Acad Sci U S A.

[187] Asai T., Adachi N., Moriya T. (2021). Cryo-EM structure of K^+^-bound hERG channel complexed with the blocker astemizole. Structure.

[188] Wu Q., Huang J., Fan X. (2023). Structural mapping of Nav1.7 antagonists. Nat Commun.

[189] Gao S., Yan N. (2021). Structural basis of the modulation of the voltage-gated calcium ion channel Cav1.1 by dihydropyridine compounds. Angew Chem Int Ed.

[190] Chen Z., Mondal A., Minor D.L. (2023). Structural basis for Ca_V_α_2_δ:gabapentin binding. Nat Struct Mol Biol.

[191] Banerjee A., Lee A., Campbell E., Mackinnon R. (2013). Structure of a pore-blocking toxin in complex with a eukaryotic voltage-dependent K^+^ channel. Elife.

[192] Tyagi A., Ahmed T., Jian S. (2022). Rearrangement of a unique Kv1.3 selectivity filter conformation upon binding of a drug. *Proc Natl Acad Sci U S A*.

[193] Zheng Y., Liu H., Chen Y. (2022). Structural insights into the lipid and ligand regulation of a human neuronal KCNQ channel. Neuron.

[194] Pan Y., Weng J., Kabaleeswaran V. (2008). Cortisone dissociates the Shaker family K+ channels from their beta subunits. Nat Chem Biol.

[195] Noble M.E., Endicott J.A., Johnson L.N. (2004). Protein kinase inhibitors: insights into drug design from structure. Science.

[196] Cohen P., Cross D., Janne P.A. (2021). Kinase drug discovery 20 years after imatinib: progress and future directions. Nat Rev Drug Discov.

[197] Callaway E. (2022). What's next for AlphaFold and the AI protein-folding revolution. Nature.

[198] Callaway E. (2023). AI tools are designing entirely new proteins that could transform medicine. Nature.

[199] Zhang K., Yang X., Wang Y. (2025). Artificial intelligence in drug development. Nat Med.

[200] Focken T., Chowdhury S., Zenova A. (2018). Design of conformationally constrained acyl sulfonamide isosteres: identification of N-([1,2,4]triazolo[4,3- a]pyridin-3-yl)methane-sulfonamides as potent and selective hNa_V_1.7 inhibitors for the treatment of pain. J Med Chem.

[201] Kschonsak M., Jao C.C., Arthur C.P. (2023). Cryo-EM reveals an unprecedented binding site for Na_V_1.7 inhibitors enabling rational design of potent hybrid inhibitors. Elife.

[202] Walters W.P. (2019). Virtual chemical libraries. J Med Chem.

[203] Melancon K., Pliushcheuskaya P., Meiler J., Kunze G. (2023). Targeting ion channels with ultra-large library screening for hit discovery. Front Mol Neurosci.

[204] Irwin J.J., Tang K.G., Young J. (2020). ZINC20—a free ultralarge-scale chemical database for ligand discovery. J Chem Inf Model.

[205] Grygorenko O.O., Radchenko D.S., Dziuba I., Chuprina A., Gubina K.E., Moroz Y.S. (2020). Generating multibillion chemical space of readily accessible screening compounds. iScience.

[206] Harris C.J., Hill R.D., Sheppard D.W., Slater M.J., Stouten P.F. (2011). The design and application of target-focused compound libraries. Comb Chem High Throughput Screen.

[207] Lavecchia A., Di Giovanni C. (2013). Virtual screening strategies in drug discovery: a critical review. Curr Med Chem.

[208] Verdonk M.L., Cole J.C., Hartshorn M.J., Murray C.W., Taylor R.D. (2003). Improved protein-ligand docking using GOLD. Proteins.

[209] Friesner R.A., Banks J.L., Murphy R.B. (2004). Glide: a new approach for rapid, accurate docking and scoring. 1. Method and assessment of docking accuracy. J Med Chem.

[210] Nguyen N.T., Nguyen T.H., Pham T.N.H. (2020). Autodock vina adopts more accurate binding poses but Autodock4 forms better binding affinity. J Chem Inf Model.

[211] Alford R.F., Fleming P.J., Fleming K.G., Gray J.J. (2020). Protein structure prediction and design in a biologically realistic implicit membrane. Biophys J.

[212] Samanta R., Gray J.J. (2024). Implicit model to capture electrostatic features of membrane environment. PLoS Comput Biol.

[213] Dominguez C., Boelens R., Bonvin A.M. (2003). HADDOCK: a protein-protein docking approach based on biochemical or biophysical information. J Am Chem Soc.

[214] Jimenez-Garcia B., Roel-Touris J., Romero-Durana M., Vidal M., Jimenez-Gonzalez D., Fernandez-Recio J. (2018). LightDock: a new multi-scale approach to protein-protein docking. Bioinformatics.

[215] Kuhn M., Firth-Clark S., Tosco P., Mey A., Mackey M., Michel J. (2020). Assessment of binding affinity via alchemical free-energy calculations. J Chem Inf Model.

[216] Lin Y., Grinter S.Z., Lu Z. (2021). Modulating the voltage sensor of a cardiac potassium channel shows antiarrhythmic effects. Proc Natl Acad Sci U S A.

[217] Hughes T.E., Del Rosario J.S., Kapoor A. (2019). Structure-based characterization of novel TRPV5 inhibitors. Elife.

[218] Yang X., Wu Y., Xu S. (2023). Targeting the inward rectifier potassium channel 5.1 in thyroid cancer: artificial intelligence-facilitated molecular docking for drug discovery. BMC Endocr Disord.

[219] Zhang G., Xu X., Jia Z. (2022). An allosteric modulator activates BK channels by perturbing coupling between Ca^2+^ binding and pore opening. Nat Commun.

[220] Oddsson S., Kowal N.M., Ahring P.K., Olafsdottir E.S., Balle T. (2020). Structure-based discovery of dual-target hits for acetylcholinesterase and the α7 nicotinic acetylcholine receptors: in silico studies and in vitro confirmation. Molecules.

[221] Gentile F., Agrawal V., Hsing M. (2020). Deep docking: a deep learning platform for augmentation of structure based drug discovery. ACS Cent Sci.

[222] Zhou G., Rusnac D.V., Park H. (2024). An artificial intelligence accelerated virtual screening platform for drug discovery. Nat Commun.

[223] Stafford K.A., Anderson B.M., Sorenson J., van den Bedem H. (2022). AtomNet PoseRanker: enriching ligand pose quality for dynamic proteins in virtual high-throughput screens. J Chem Inf Model.

[224] Atomwise AIMS Program (2024). AI is a viable alternative to high throughput screening: a 318-target study. Sci Rep.

[225] Zeng X., Wang F., Luo Y. (2022). Deep generative molecular design reshapes drug discovery. Cell Rep Med.

[226] Colby S.M., Nunez J.R., Hodas N.O., Corley C.D., Renslow R.R. (2020). Deep learning to generate in silico chemical property libraries and candidate molecules for small molecule identification in complex samples. Anal Chem.

[227] Schultz K.J., Colby S.M., Yesiltepe Y., Nunez J.R., McGrady M.Y., Renslow R.S. (2021). Application and assessment of deep learning for the generation of potential NMDA receptor antagonists. Phys Chem Chem Phys.

[228] Liu X., Ye K., van Vlijmen H.W.T., IJzerman A.P., van Westen G.J.P. (2023). DrugEx v3: scaffold-constrained drug design with graph transformer-based reinforcement learning. J Cheminform.

[229] Kalia J., Milescu M., Salvatierra J. (2015). From foe to friend: using animal toxins to investigate ion channel function. J Mol Biol.

[230] Norton R.S., Pallaghy P.K. (1998). The cystine knot structure of ion channel toxins and related polypeptides. Toxicon.

[231] Pennington M.W., Czerwinski A., Norton R.S. (2018). Peptide therapeutics from venom: current status and potential. Bioorg Med Chem.

[232] King G.F. (2011). Venoms as a platform for human drugs: translating toxins into therapeutics. Expert Opin Biol Ther.

[233] Robinson S.D., Undheim E.A.B., Ueberheide B., King G.F. (2017). Venom peptides as therapeutics: advances, challenges and the future of venom-peptide discovery. Expert Rev Proteomics.

[234] Schmalhofer W.A., Calhoun J., Burrows R. (2008). ProTx-II, a selective inhibitor of Na_V_1.7 sodium channels, blocks action potential propagation in nociceptors. Mol Pharmacol.

[235] Park J.H., Carlin K.P., Wu G. (2014). Studies examining the relationship between the chemical structure of protoxin II and its activity on voltage gated sodium channels. J Med Chem.

[236] Bender B.J., Cisneros A., Duran A.M. (2016). Protocols for molecular modeling with Rosetta3 and RosettaScripts. Biochemistry.

[237] Kuyucak S., Norton R.S. (2014). Computational approaches for designing potent and selective analogs of peptide toxins as novel therapeutics. Future Med Chem.

[238] Rashid M.H., Kuyucak S. (2014). Free energy simulations of binding of HsTx1 toxin to Kv1 potassium channels: the basis of Kv1.3/Kv1.1 selectivity. J Phys Chem B.

[239] Ngo K., Lopez Mateos D., Han Y. (2024). Elucidating molecular mechanisms of protoxin-II state-specific binding to the human NaV1.7 channel. J Gen Physiol.

[240] Nguyen P.T., Harris B.J., Mateos D.L., Gonzalez A.H., Murray A.M., Yarov-Yarovoy V. (2024). Structural modeling of ion channels using AlphaFold2, RoseTTAFold2, and ESMFold. Channels.

[241] Jumper J., Evans R., Pritzel A. (2021). Highly accurate protein structure prediction with AlphaFold. Nature.

[242] Baek M., DiMaio F., Anishchenko I. (2021). Accurate prediction of protein structures and interactions using a three-track neural network. Science.

[243] Watson J.L., Juergens D., Bennett N.R. (2023). De novo design of protein structure and function with RFdiffusion. Nature.

[244] Pacesa M., Nickel L., Schellhaas C. (Published online August 27, 2025). One-shot design of functional protein binders with BindCraft. Nature.

[245] Vazquez Torres S., Leung P.J.Y., Venkatesh P. (2024). De novo design of high-affinity binders of bioactive helical peptides. Nature.

[246] Lopez-Mateos D., Harris B.J., Hernandez-Gonzalez A., Narang K., Yarov-Yarovoy V. (2025). Harnessing deep learning methods for voltage-gated ion channel drug discovery. Physiology.

[247] Usher S., Hink F., Sörmann J., Rogers J., Pless S.A. (2025). BPS2025-Diffusing protein binders to plug the sodium leak. Biophys J.

[248] Yehya M., Mahling R.W., Fossier L., Jayaraman S., Kochiss A.L., Ben-Johny M. (2025). BPS2025—A de novo designed miniprotein prevents Ca2+-feedback of CaV1 channels. Biophys J.

[249] Narang K., Mateos D.L., Murray A., Yarov-Yarovoy V. (2025). BPS2025—Computational design of Na_V_1.8 sodium channel inhibitors as novel non-addictive treatments for pain management. Biophys J.

[250] Mahling R., Hegyi B., Cullen E.R. (2025). De novo design of a peptide modulator to reverse sodium channel dysfunction linked to cardiac arrhythmias and epilepsy. Cell.

[251] Abramson J., Adler J., Dunger J. (2024). Accurate structure prediction of biomolecular interactions with AlphaFold 3. Nature.

[252] Wohlwend J, Corso G, Passaro S, et al. Boltz-1 democratizing biomolecular interaction modeling. Preprint. Posted online May 6, 2025. bioRxiv 624167. doi:10.1101/2024.11.19.624167.

[253] Bennett NR, Watson JL, Ragotte RJ, et al. Atomically accurate de novo design of antibodies with RFdiffusion. Preprint. Posted online February 28, 2025. bioRxiv 585103. doi:10.1101/2024.03.14.585103.

[254] Rettie S.A., Juergens D., Adebomi V. (Published online June 20, 2025). Accurate de novo design of high-affinity protein binding macrocycles using deep learning. Nat Chem Biol.

[255] Vinogradov A.A., Yin Y., Suga H. (2019). Macrocyclic peptides as drug candidates: recent progress and remaining challenges. J Am Chem Soc.

[256] Johns D.G., Campeau L.C., Banka P. (2023). Orally bioavailable macrocyclic peptide that inhibits binding of PCSK9 to the low density lipoprotein receptor. Circulation.

[257] Bissonnette R., Pinter A., Ferris L.K. (2024). An oral interleukin-23-receptor antagonist peptide for plaque psoriasis. N Engl J Med.

[258] Zhou Y., Zhang W., Sun F. (2025). Acoltremon: the first TRPM8 agonist approved for the treatment of dry eye disease. Drug Discov Ther.

[259] Xu Z., Ren F., Wang P. (2025). A generative AI-discovered TNIK inhibitor for idiopathic pulmonary fibrosis: a randomized phase 2a trial. Nat Med.

[260] Zitnik M. (2025). AI-enabled drug discovery reaches clinical milestone. Nat Med.

